# Adaptation of *Coccomyxa* sp. to Extremely Low Light Conditions Causes Deep Chlorophyll and Oxygen Maxima in Acidic Pit Lakes

**DOI:** 10.3390/microorganisms8081218

**Published:** 2020-08-11

**Authors:** Javier Sánchez-España, Carmen Falagán, Diana Ayala, Katrin Wendt-Potthoff

**Affiliations:** 1 Spanish Geological Survey, Geochemistry and Sustainable Mining Unit, Calera 1, Tres Cantos, 28760 Madrid, Spain; 2 Environmental and Sustainability Institute and Camborne School of Mines, University of Exeter, Penryn Campus, Penryn, Cornwall TR10 9FE, UK; c.falagan@exeter.ac.uk; 3 Department of Civil and Environmental Engineering, The Pennsylvania State University, 212 Sackett Building, University Park, PA 16802, USA; dka9@psu.edu; 4 Helmholtz Centre for Environmental Research—UFZ, Brückstraße 3a, 39114 Magdeburg, Germany; katrin.wendt-potthoff@ufz.de

**Keywords:** acidophiles, deep chlorophyll maxima, green algae, phytoplankton, photosynthetically active radiation, dissolved oxygen, primary production, *Coccomyxa* sp.

## Abstract

Deep chlorophyll maxima (DCM) and metalimnetic oxygen maxima (MOM) are outstanding biogeochemical features of acidic pit lakes (APL). However, knowledge of the eukaryotic phototrophs responsible for their formation is limited. We aimed at linking the dynamics of phototrophic communities inhabiting meromictic APL in Spain with the formation of these characteristic layers. Firstly, the dynamics of DCM and MOM and their relation to physico-chemical parameters (photosynthetically active radiation (PAR), pH, dissolved ferric iron concentration, temperature), pigments and nutrient distribution is described; secondly, the phototrophic community composition is studied through a combination of microscopy, biomolecular and “omics” tools. Phototrophic communities of the studied APL show a low diversity dominated by green microalgae, specifically *Coccomyxa* sp., which have been successfully adapted to the chemically harsh conditions. DCM and MOM are usually non-coincident. DCM correspond to layers where phototrophs have higher chlorophyll content per cell to cope with extremely low PAR (<1 µmol m^−2^ s^−1^), but where photosynthetic oxygen production is limited. MOM correspond to shallower waters with more light, higher phytoplankton biomass and intense photosynthetic activity, which affects both oxygen concentration and water temperature. The main drivers of DCM formation in these APL are likely the need for nutrient uptake and photo-acclimation.

## 1. Introduction

Deep chlorophyll maxima (DCM) are subsurface layers enriched in chlorophyll and located at certain depths below the surface of thermally stratified water bodies such as lakes or the open ocean [1,2,3,4,5]. DCM are usually formed at depths where nutrient availability (usually increasing with depth) and light intensity (decreasing with depth) are compensated [2]. Photosynthetic activity has a big effect on the biogeochemistry of the water column, providing organic carbon for other microorganisms, sustaining other planktonic organisms and altering oxygen concentrations in the water column of freshwater and oceanic environments. When the production of oxygen surpasses consumption, it can generate a metalimnetic oxygen maxima (MOM) that can reach values above 100% saturation. DCM and MOM can co-occur, especially when DCM are shallow [6]; but both maxima can be also found at different depths, as is commonly the case when DCM are deeper and the MOM is generally situated above it [6]. In addition to the biological (photosynthetic) activity, there are certain physical factors such as temperature which may also contribute to the formation of the MOM, representing as much as 60% of the MOM, as in certain lakes of Wisconsin and Michigan, USA [7]. In a comprehensive study on the patterns and drivers of DCM in 100 seasonally stratified lakes around the world, Leach et al. (2017) [3] have recently concluded that the light attenuation (e.g., attenuation coefficient or 1% photosynthetically active radiation (PAR) depth, both depending mostly on dissolved organic carbon concentration) was a more reliable predictor of DCM depth than thermal stratification (e.g., thermocline slope and depth). The authors also noted that the DCM thickness could be best described by the lake size, with larger lakes displaying thicker DCM. Finally, Leach et al. (2017) [3] found that DCM are usually dominated by diatoms, dinoflagellates, chrysophytes, cryptophytes, and less frequently, cyanobacteria, as all these groups can maintain their vertical position in the water column through active swimming or buoyancy regulation, by decreased settling rate under nutrient-depleted conditions, or by increased growth rate under low light conditions due to the presence of phycoerythrin [3]. A detailed revision on the occurrence and ecology of DCM has also been provided for Spanish lakes [5], though this study focused on freshwater lakes neutral to alkaline in mountain regions or karstic environments (saline to hypersaline) where cryptophytes and/or purple sulfur bacteria, cyanobacteria, and anoxygenic phototrophs are usually the dominating microorganisms forming the DCM. A revision of the different mechanisms that have been proposed so far to explain the formation of DCM in freshwater lakes and reservoirs is reported in Table 1.

In comparison with their circumneutral counterparts, fewer studies exist on DCM formed in acidic lakes. Examples include the acidic volcanic crater lake Caviahue in Patagonia (Argentina) [14], or the acidic mine pit lake ML111 in the Lusatian mining district (Germany). A major difference with respect to freshwater lakes is that light attenuation in acidic lakes is mostly caused by absorption by Fe(III) and not by dissolved organic matter, which tends to be photochemically degraded at low pH [15]. Nutrient availability and light attenuation by Fe(III) were found to be the main contributors to the formation of the DCM in the Patagonian lake [14]. In lake ML111, DCM was found to be the result of *Chlamydomonas (C.) acidophila* concentrating in deeper layers in order to increase the uptake of inorganic carbon and phosphorus [16,17], and also to avoid grazing by *Ochromonas* spp. in upper layers [18]. A recent study has investigated the nature and dynamics of a DCM formed at a depth of 20 m in an acid mine drainage (AMD)-impacted reservoir in Southwestern Spain [19], where the chlorophyte *Carteria* spp. was found to be dominant, and where the need of CO_2_ uptake has been proposed as driving mechanism to explain the concentration of this phototroph. The development and significance of MOM in acidic lakes is still less commonly documented and far less understood, despite the fact that these deep oxygen peaks may have a profound effect on the occurrence and kinetics of certain bacterial metabolisms such as those of Fe(II) or reduced sulfur oxidation. The development of DCM and MOM in these acidic systems and the interrelation between these two biogeochemically important layers is therefore of major scientific interest.

In the field of pit lake research, the case of the Iberian Pyrite Belt (IPB) mining district is paradigmatic since it contains more than 20 pit lakes formed by the flooding of former metal sulfide mines [20,21]. These pit lakes are highly acidic (pH mostly between 2.0 and 3.0) as a result of intensive mineral dissolution (mostly pyrite and other sulfides), and display high concentrations of many different metals, including Fe, Al, Mn, Cu or Zn, in addition to many other toxic trace elements such as As, Cr, Cd, Pb, Co or Ni [20,21] (Table 2, Table S1). The majority of pit lakes in the IPB are meromictic (i.e., they display permanent stratification with an upper, oxidizing mixolimnion and a lower, anoxic and reducing monimolimnion [22,23]). Despite their extreme chemical conditions, many of these APL show seasonal development of DCM and MOM [23]. Recent research has shown the critical importance of these DCM to promote and sustain anaerobic bacterial metabolisms (e.g., sulfate and iron reduction) below the redoxcline of the APL by supplying fresh organic carbon (exudates and lysates released during decomposition of settling phytoplanktonic biomass) [24,25]. The study by Diez-Ercilla et al. (2014) [24] demonstrated that the position and intensity of DCM are critical factors controlling the extent of sulfate reduction and biogenic H_2_S production in the redoxcline by microbes like *Desulfomonile* spp. [25]. This has led to propose artificial eutrophication through addition of nutrients (e.g., phosphate, ammonium) to enhance phytoplankton growth and indirectly promote sulfate reduction below the redoxcline as an efficient and cost-effective mechanism of remediation in these pit lakes [24]. However, very little is still known with regard to the occurrence and formation of DCM and MOM in these pit lakes. Essential aspects that remain unknown include: (i) the phototrophs responsible for the formation of the DCM, (ii) the relation between DCM and MOM in these particular lakes, (iii) the mechanisms responsible for the formation of DCM and MOM, and (iv) their relation to physical and chemical parameters. A comprehensive study of these systems is necessary to understand the biological controls on the chemistry of the APL in future remediation strategies. The main objective of this manuscript was therefore to shed light to these questions and expand the current knowledge on DCM and MOM formation in lakes in a general sense (Table 1) and in acidic, metal-rich lakes in particular. For this purpose, we have selected several APL in Spain (Figure S1). We were particularly interested in deciphering the relation of the studied DCM and MOM to key physico-chemical variables such as water temperature, light and nutrient availability, and ferric iron concentration. We also aimed at linking the distribution and seasonal evolution of DCM and MOM with data on algal biomass and pigment concentrations. The working hypotheses of our study were (1) DCM formation is mainly driven by light and nutrients, (2) the phototrophic community is composed by specific organisms differing from those of other acidic waters, and (3) these phototrophs display spatial and seasonal adaptations to their extreme habitat. Our study will help to expand the current knowledge on the adaptive capacity of acidophilic phototrophs to extremely acidic, metal-rich and low light environments.

## 2. Materials and Methods

### 2.1. Site Description

Three meromictic APL that have been investigated during the last decade (2009–2019): Cueva de la Mora (CM; the most intensively studied APL in Spain), Herrerías-Guadiana (HER) and San Telmo (ST) were chosen for this study. Some basic geometric and physico-chemical data of these pit lakes are provided in Table 2, and their geographic location and satellite images are given in Figure S1. Further information about the limnology, chemistry and bacterial ecology of these pit lakes can be found in previous papers by the authors [20,21,22,23,24,25,26,27]. Sparse data (e.g., profiles of chlorophyll-a, dissolved oxygen (DO), temperature or PAR intensity) collected in other Spanish APL (i.e., Brunita pit lake, near Cartagena, Southeastern Spain [28]; Barruecopardo, near Salamanca, Northwestern Spain) have been included to complete the discussion about (i) photosynthetic oxygen production, (ii) thermal effects associated with the MOM, and (iii) diurnal evolution of the phytoplankton communities.

### 2.2. In Situ Measurements

Chlorophyll-a (Chl-a) concentration, PAR, dissolved oxygen (DO) and water temperature (T) were measured with multiparametric datasondes (MS5 and DS5 from Hydrolab, Hach, Loveland, CO, USA). These profiles were always taken during the early morning to ensure a comparable dataset for all lakes. Measurements were generally started at 0.5 m below the lake surface and were manually recorded at depth intervals of between 0.5 and 1 m across the photic zone (generally the upper 10 m) or until the total extinction of all these parameters. CM sampling took place in February 2007 and 2009, March, July and November 2010, July and September 2011 and March 2012; HER in March 2008 and April, July and September 2011; San Telmo in April and July 2011; Brunita in November 2018; and Barruecopardo in January 2008. The DS5 datasonde was equipped with a fluorometer and a Chl-a Hach sensor, which transforms relative fluorescence units to Chl-a concentration in µg/L [29]. The measuring range of this Chl-a sensor was 0–50 µg/L, the accuracy was ± 3% for signal level equivalents of 1 ppb rhodamine water tracer (WT) dye, and the resolution was 0.01 µg/L. The conversion of fluorescence signal to Chl-a concentration is instrument-specific and may vary with respect to other sensors developed by other manufacturers. PAR was measured with a spherical underwater quantum sensor from LI-COR Biogeosciences (Lincoln, NE, USA). This sensor includes a radiometer coupled to a probe (measuring range of 0 to 10,000 µmol s^–1^ m^–2^, accuracy of ± 5% of reading or ± 1 µmol s^–1^ m^–2^, and resolution of 1 µmol s^–1^ m^–2^). The vertical light attenuation coefficient (η) and the depth for 1% PAR (Z_1%_) were calculated based on the PAR profiles. The vertical light attenuation coefficient is the percentage of the surface light absorbed or scattered in a 1-m-long vertical column of water, whereas Z_1%_ is the depth of the lake where PAR intensity is exactly 1% of the PAR value measured at the lake surface. DO was measured by luminescence with a Hach sensor (measuring range: 0–30 mg/L O_2_, accuracy: ±0.01 mg/L for 0–8 mg/L and ±0.02 mg/L for > 8 mg/L). D-Opto loggers (Zebra-Tech, LTD, Nelson, New Zealand) were also installed in CM to record DO concentration at several depths through the photic zone during a cycle of 3 days in September 2011; the measuring range of these loggers was 1% of measurement or 0.02 ppm O_2_, and their resolution was 0.01% saturation or 0.001 ppm O_2_.

### 2.3. Field Sampling

Sampling of waters for microscopic study of phytoplanktonic microorganisms, nutrient analyses, pigment analyses, and metagenomic and metatranscriptomic analyses was conducted with a Van Dorn sampling bottle (KC Denmark A/S, Silkeborg, Denmark) with 5 L capacity. A detailed (cm-scale) sampling was also performed through the redoxcline of CM in September 2011 with a syringe sampler designed for this purpose [30]. Water samples (1–2 L) for pigment analyses were collected in July and September in 2011. Samples were filtered on site through 0.7 μm pore-size glass fiber filters GF/F Whatman and stored at −20 °C until further analysis. Unfiltered water samples for counting and measuring microbial cells were fixed on site with Lugol’s solution or formaldehyde (final concentration 4%) and stored at 4 °C. Duplicate samples for metagenomic and metatranscriptomic analyses of DNA and RNA were collected in May 2018 from the MOM of CM (situated at 3 m depth) and from the chemocline (at 11 m depth) using the abovementioned Van Dorn sampling bottle. One liter for RNA extraction and up to 3 L for DNA extraction were pre-filtered through 100 µm and then filtered through 0.22 µm pore-size Sterivex^TM^ filter units on site (Figure S2a). The filtration of these volumes of water with the Sterivex^TM^ filters, as well as with other types of filters used in standard (e.g., 0.45 µm) filtration, always resulted in the accumulation of substantial phototrophic microbial biomass as evidenced by the intense green color of the filters denoting the presence of abundant chlorophyll (Figure S2). The filters were always preserved in dark conditions and stored in dry ice at −80 °C until processed.

### 2.4. Nutrient and Iron Concentration, Pigment Analyses and Cell Counts

Nutrient concentrations, including nitrate- and ammonium-nitrogen, phosphate-phosphorus, and dissolved inorganic carbon (DIC, given as CO_2_) were analyzed with a portable UV-VIS DR2800 spectrophotometer (Hach, Loveland, CO, USA) using cuvette tests LCK 339 (NO_3_-N, 0.23–13.50 mg/L), LCK 304 (NH_4_-N, measurement range of 0.05–2.0 mg/L), LCK 348 (PO_4_-P, 0.05–5.0 mg/L), and LCK 388 (CO_2_, 55–550 mg/L), respectively, following analytical procedures by Hach Lange GmbH [31]. The automatic routine conducted 10 analyses per sample and the values given here correspond to the mean value of these 10 measurements. Duplicates were also analyzed in certain campaigns (e.g., August 2009 in CM, July 2019 in BRU) and a good reproducibility was obtained in all cases. Dissolved silica (SiO_2_) and dissolved total iron (Fe_t_) were measured by inductively coupled plasma-atomic emission spectrometry (ICP-AES) using a Varian Vista-MPX. The speciation of iron (Fe[II]/Fe[III]) was further assessed by colorimetric reflectance photometry with an RQflex10 reflectometer (MERCK KGaA, Darmstadt, Germany) and Reflectoquant analytical strips (MERCK KGaA) [26]. This method measures Fe(II) by two different colorimetric reactives (Ferrospectral –a triazine derivative– for Fe(II) < 358 µM and 2.2′-bipiridine for Fe(II) > 358 µM), showing distinct analytical resolution depending on the aqueous Fe(II) concentration range (±1.8 µM in the 8.95–358 µM range, and ±17.9 µM in the 358–3580 µM range). The detection limit of the analytical technique is 8.95 µM (0.5 mg/L Fe[II]). The Fe(II) determinations were firstly conducted in intact fresh samples immediately after collection and then after addition of ascorbic acid, which reduces all Fe(III) to Fe(II). The amount of Fe(III) in the samples is then calculated by difference (Fe[III]=Fe_t_ – Fe[II]). In oxidized water samples of the mixolimnion, like the ones included in this study, total iron is entirely composed of Fe(III). Pigment extraction was performed at room temperature. Biomass containing glass fiber filters was washed with neutralized acetone to avoid pigment reduction. Acetone neutralization was achieved by passing it throughout MgCO_3_. Samples were stored in darkness for 24 h at 4 °C to allow the pigment extraction to occur. Pigment composition was determined in the University of Cádiz (Department of Biology, Faculty of Marine and Environmental Sciences, Puerto Real, Spain) and in the UFZ lab at Magdeburg, with spectrophotometer scanning at absorbance from 300 to 900 nm (University of Cádiz) or by HPLC (UFZ). In the former case, a smoothing running average with a window of five nm was performed (using MS Excel) on pigment spectra in order to remove high frequency variability. Individual cells of phytoplanktonic microorganisms were estimated in some campaigns (July and September 2011, September 2017) following the Utermöhl method or staining with acridine orange and fluorescence microscopy.

### 2.5. DNA and RNA Analyses

DNA and RNA extractions were carried out using the Qiagen DNAeasy Powerwater Kit and the Qiagen RNeasy PowerMicrobiome Kit, respectively (Qiagen, Venlo, Netherlands). Quantification of DNA and RNA was carried out using Qubit^®^ 2.0 Fluorometer (Invitrogen, Carlsbad, CA, USA), and RNA was also quantified with the Bioanalyzer 2100 RNA 6000 pico Assay (Agilent, Santa Clara, CA, USA). Metagenome library preparation was performed using Illumina’s NexteraXT library preparation kit (Illumina, San Diego, USA) with 1 ng of total genomic DNA as starting material, tagmented with Illumina adapters and unique 8 bp dual indices, and 12 PCR cycles. Double-stranded cDNA synthesis and metatranscriptome library preparation was performed using the Tecan RNA Trio library preparation kit (Tecan, Mannedorf, Switzerland) with 10 µL of RNA as starting material and two rounds of PCR, the first one with 6 PCR cycles and the second one with 8 PCR cycles. Metagenome libraries were normalized and multiplexed using the molarity between 250–400 bp. Metatranscriptome libraries were also normalized and multiplexed as the metagenome ones. Libraries were then purified on a 2% agarose gel and size selected using the QIAquick gel extraction kit (Qiagen, Venlo, Netherlands). Sequencing was conducted on an Illumina Hiseq 4000 platform (Illumina, San Diego, CA, USA) using 150 bp paired end chemistry.

Raw metagenomic data (available in https://www.ncbi.nlm.nih.gov/sra/PRJNA646106) were quality controlled and trimmed before de novo assembly with Megahit v1.1.2 [32]. Prodigal v2.6.3 [33] and MetaEuk [34] were used to predict prokaryotic and eukaryotic protein-encoding genes for each metagenome, respectively. Taxonomic annotation of the predicted genes was conducted with DIAMOND v0.9.32.133 [35] against the NCBI-nr database (Coordinators 2016, Database resources of the National Center for Biotechnology Information), (50% identity over at least 80% length). The top hits per predicted gene were retrieved with Megan v6.18.10 [36]. GhostKOALA [37] was also used for taxonomic annotation of predicted genes. Priority was given to the taxonomic assignation provided by DIAMOND+Megan, but for those not assigned, the assignations provided by GhostKOALA were considered. Functional annotation was conducted with KOFAMscan v1.3.0 [38] and the KO+Pfam database released on May 2020. DNA-RPKM values (reads mapped to a gene per kilobase of the gene per total million mapped reads) were calculated for each predicted gene using BBMap [39]. Quality-filtered metatranscriptomes were used after in-silico rRNA depletion of raw data using SortMeRNA v2.1 [40]. The mRNA reads were mapped to the respective metagenome contigs with BBMap [39] to calculate RNA-RPKM values.

## 3. Results and Discussion

### 3.1. Light Attenuation by Dissolved Fe(III)

**Figure 1:**
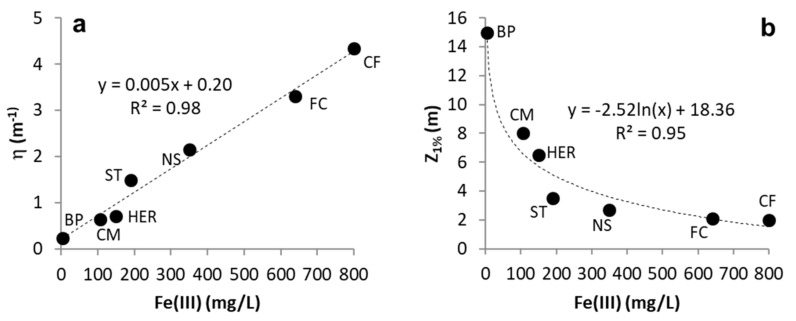
Binary plots showing dependence of light attenuation coefficient (η) (**a**) and depth of 1% surface light (Z_1%_) (**b**) in different APL with respect to dissolved ferric iron concentration (Fe(III)). The regression lines (linear or exponential) and corresponding correlation coefficients are indicated in both cases. The error bars (based on standard deviation of analytical Fe(III) determinations) lie within the same data points. Pit lake abbreviations: BP, Barruecopardo; CM, Cueva de la Mora; HER, Herrerías; ST, San Telmo; NS, Nuestra Señora del Carmen; FC, Filón Centro (Tharsis); CF, Confesionarios.

**Figure 2:**
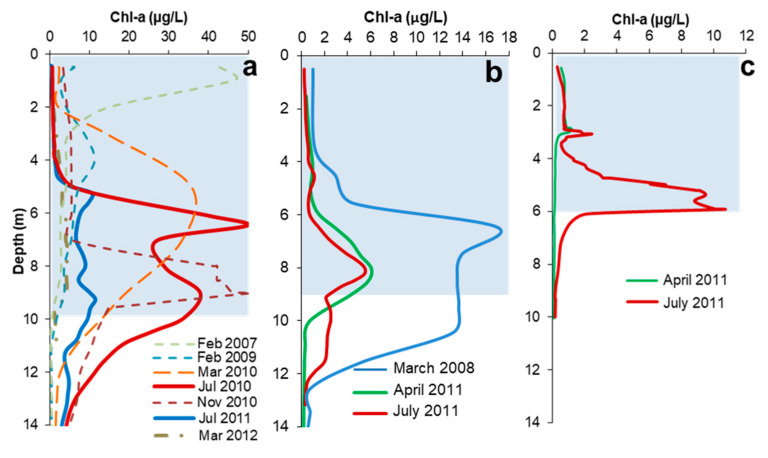
Vertical profiles of chlorophyll-a (Chl-a) concentration across the photic zone of three acidic pit lakes in Southwestern Spain in different seasons: Cueva de la Mora (a), Herrerías (b) and San Telmo (c). The three profiles have been set to 14 m for comparison purposes. The photic zone (light blue colored area in the three profiles) varies between these pit lakes (e.g., 6 m in ST vs. 9–10 m in CM and HER; see also Figure 4a for a comparison of PAR profiles).

A clear relation between Fe(III) concentration and the attenuation coefficient (η) is evident in the studied pit lakes (Figure 1). Using data from several pit lakes (*n* = 7), the Fe(III) content appeared highly correlated with the attenuation coefficient (Figure 1a) and with Z_1%_ (Figure 1b). The lake with the lowest Fe(III) concentration (3.5 mg/L), lowest attenuation coefficient (0.23 m^−1^) and thickest photic zone (16 m) is Barruecopardo, whereas the lake with the highest Fe(III) concentration (>800 mg/L), highest attenuation coefficient (4.34 m^−1^) and narrowest photic zone (2 m) is Confesionarios. In the latter, PAR is virtually extinct below 2 m and only a very small peak of Chl-a concentration has been measured [21]. On the other hand, in the pit lakes with lowest Fe(III) concentration and lowest extinction coefficients, such as Barruecopardo, CM, or Concepción, deeper and thicker DCM are usually observed (DCM of CM is shown in Figure 2).

These findings match with previous studies reporting that, unlike freshwater lakes (where DOC is the most important factor controlling light attenuation), ferric iron (Fe(III)) is what adsorbs most of the PAR radiation in APL [15]. In addition, Fe(III) filters notably the wavelength of the little light reaching the deep layers, and only that corresponding to the red spectrum (which is not suitable for photosynthesis for most green algae and other phytoplankton) reaches deeper waters [5]. This might contribute to further decreasing the biodiversity of eukaryotic microorganisms (already biased by the acidic conditions) living in deeper layers, as they have to cope not only with very low light, but also with a very specific type of light (red) which is not so effective for photosynthesis by reaction with Chl-a [5].

Another question related to the attenuation of solar radiation refers to the absorption of UV light. In lakes with extremely clear waters (e.g., Alpine mountain lakes), the penetration of UV radiation may be important enough as to represent a problem for phytoplankton causing photo-inhibition or cell damage [5]. In the studied APL, the dose and intensity of UV radiation in the lake surface in the central hours of the day in summer can be very high (e.g., up to 30 W/m^2^ [26]), but the attenuation of UV-A light is so strong (due to the interaction of photons with Fe(III) ions), that it is totally absorbed in the first few centimeters (5–6 cm in most lakes, 0.5 cm in Confesionarios [26]). Therefore, UV radiation is unlikely to influence DCM formation in the studied APL.

### 3.2. General Patterns of DCM Formation and Pigment Distribution

Vertical profiles of Chl-a concentration obtained with the fluorimetric sensor in different seasons in three pit lakes (CM, HER, ST) are displayed in Figure 2. These profiles show a general pattern with DCM occurring at depths between 3 and 8–9 m, depending on the pit lake and the observation period. The Chl-a profiles also evidence that DCM are usually not single, but complex (double, or even triple) multi-peak profiles. At first sight, there is no apparent temporal pattern (e.g., spring bloom), and DCM may occur throughout a big part of the year. Only in the winter period (December to February) when water temperature is coldest (10–12 °C, not shown) are DCMs actually absent. During winter, the mixolimniom is fully homogenized (unlike the warmer months when the mixolimniom is stratified), and phytoplankton is usually concentrated near the lake surface (see February 2007 profile in Figure 2a). In the particular case of CM, however, a temporal evolution with progressive deepening of the DCM appeared evident in 2010 and 2011 (Figure 2a). The Chl-a profiles showed a wide peak in March 2010 at 5 m depth, with a further development of two sub-peaks (6 and 9 m) in July of the same year. The existence of different chlorophyll peaks in a given profile has been classically interpreted as representing different populations of photosynthetic microorganisms adapted to different conditions [1], although in this study no definitive evidence to sustain this hypothesis has been found. A deep Chl-a maximum was also detected between 8 and 9 m in November 2010.

The maximum Chl-a concentrations observed in the DCM formed in the three pit lakes vary in intensity, from the lowest value of 10 µg/L observed in ST (Figure 2c) to the highest values around 50 µg/L found in CM (Figure 2a). This is well correlated with the Fe(III) concentrations (Figure 1); the pit lake showing the highest concentration of Fe(III) (ST, 202 mg/L Fe(III); Table 2) displays the least intense Chl-a peaks, and the pit lake showing the lowest Fe(III) content (CM, 108 mg/L Fe(III); Table 2) exhibits the most intense peaks. As previously stated, dissolved Fe(III) controls the water transparency and light penetration at depth, which in turn controls the depth at which DCM can occur. A detailed observation of the CM profiles allows to conclude that the seasonal dynamics of the DCM does not follow a simple pattern. Thus, in the same lake, the Chl-a profiles obtained in similar seasons can be rather different from one another (see, for example, differences between July 2010 and July 2011, or March 2010 vs. March 2012; Figure 2a). This fact suggests that factors other than water temperature or PAR intensity (e.g., nutrient availability) may also control the phytoplankton dynamics through the water column.

**Figure 3:**
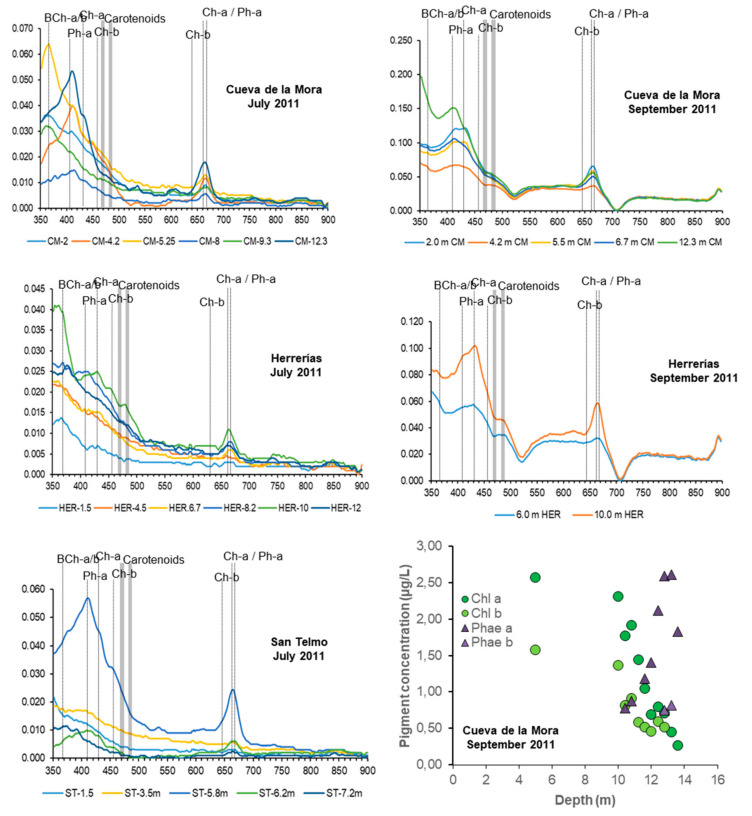
Absorbance spectra of pigments obtained for different depths (indicated by contour lines of different color in every plot) in the acidic pit lakes of Cueva de la Mora (CM; top), Herrerías (HER; center) and San Telmo (ST; left bottom). Analyses were conducted on water samples taken in July and September 2011. The position of different pigment peaks (Ch-a, chlorophyll-a; Ch-b, chlorophyll-b; BCh-a/b, bacteriochlorophyll- and –b; Ph-a, phaeophytin-a; carotenoids) are indicated in all cases. Spectra were smoothed using a 5 nm running average. Right bottom: Pigment concentration (Chl-a, Chl-b, Phae-a, Phae-b) measured in Cueva de la Mora in September 2011.

Pigment analyses conducted in 2010 and 2011 (Figure 3) at different depths in the photic zone and in the chemocline of CM, HER and ST showed that the pigments varied not only vertically, but also seasonally (e.g., July to September). Chl-a and phaeophytin-a were the main contributors to the bulk pigment composition. The latter was dominant at 12.3 m in CM, which corresponds with the deepest Chl-a maximum detected in July and September 2011. Interestingly, bacteriochlorophyll-a peaks were detected in CM at depths of 5 to 8 m and in HER at depths of 8 to 10 m in July 2011 but seemed absent in September 2011 (Figure 3). Carotenoids were also detected in most of the samples, although less evident than other pigments.

The vertical distribution of four different pigments (chlorophyll-a, chlorophyll-b, phaeophytin-a and phaeophytin-b) in CM in September 2011 is also shown in Figure 3. Phaeophytin-a concentration and Chl-a concentration followed opposite trends, which illustrates the conversion of chlorophyll to phaeophytin by degradation during settling of senescent algal cells. The high phaeophytin-a concentrations detected at 13 m are fully coincidental with the deepest Chl-a peak obtained on site with the fluorometric sensor (see also Figure 6a and Figure 7b in a later section). This observation suggests that, in agreement with previous studies [41], some of the deepest peaks observed in multi-peaked profiles of Chl-a, as revealed by fluorescence signal, are very likely the result of the accumulation of senescent algal cells in an advanced state of degradation as they reach the denser, aphotic and anoxic zone below the redoxcline. The algal debris represents the main organic substrate for heterotrophic iron- and sulfate-reducing bacteria inhabiting the redoxcline [24,25].

### 3.3. Relation of DCM with PAR

**Figure 4:**
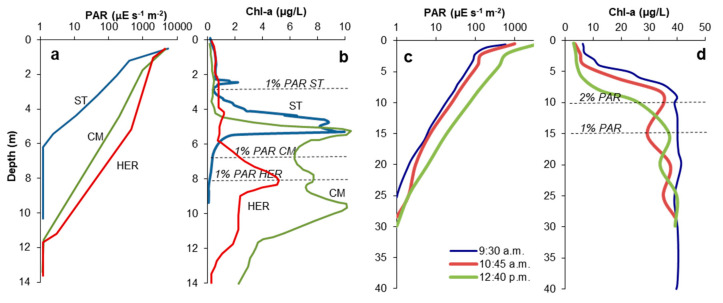
(**a**,**b**) Comparison of representative summer profiles of PAR (**a**) and chlorophyll-a concentration (**b**) in the APL of Cueva de la Mora (CM), Herrerías-Guadiana (HER) and San Telmo (ST) (all of them were taken in July 2011). (**c**,**d**) Diurnal variation of PAR (**c**) and Chl-a concentration (**d**) in Barruecopardo, as measured in January, 2008. 1% PAR = depth at which PAR is 1% of the lake surface.

**Figure 5:**
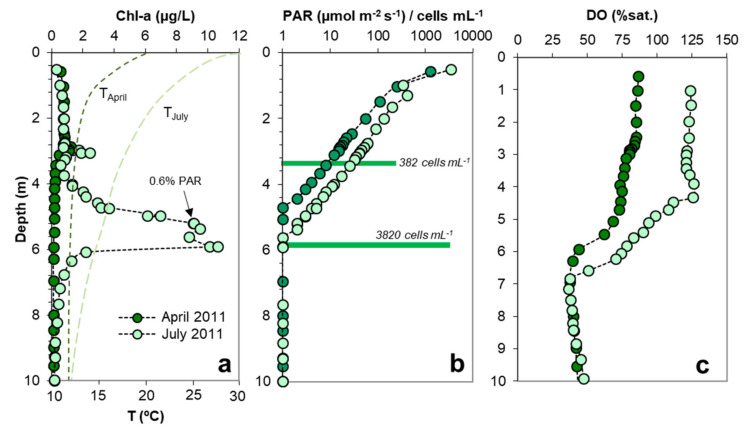
Depth profiles of chlorophyll-a concentration (**a**), PAR (**b**) and DO concentration (**c**) across the photic zone (upper 10 m) of San Telmo APL in two different seasons. The horizontal green bars in (b) indicate the total number of cell counts of phytoplanktonic phototrophs at depths of 3.5 and 6 m.

Representative profiles of PAR and Chl-a taken in CM, HER and ST are shown in Figure 4. The photic zone is 5–6 m thick in ST, and 9–10 m in CM and HER. It may be inferred that the stronger the attenuation of PAR is, the shallower the DCM is formed. The depth for 1% PAR (Z_1%_ in Figure 1) could even be used as a proxy to predict the approximate position of the upper Chl-a peak in these pit lakes (Figure 4b). There are, however, DCMs situated below this reference depth in all three pit lakes (Figure 4b). In ST, DCM exist at depths where PAR intensities are as low as 1 µmol m^−2^ s^−1^, which represents around 0.03% of the radiation reaching the lake surface (Figure 5a,b).

This varying PAR intensity could also influence the dynamics of DCM formation at a diurnal basis (Figure 4c,d). In Barruecopardo, the increase of PAR during the morning of a clear winter day, reflected in available solar light reaching deeper waters, led to important decreases of Chl-a concentration in the upper 10 m of the water column. Considering reported velocities of displacement by motile eukaryotic cells through the water column of lakes [42], it is unlikely that populations could migrate downwards over distances of several meters in a short interval of three hours. In this sense, temporal photo-inhibition of phytoplanktonic species situated in the upper waters seems a more reasonable explanation (see Section 3.9).

The shape of the Chl-a profiles and the depth of the DCM differ markedly between spring and summer of the same year, as shown for ST in 2011 (Figure 5). In this pit lake, the small Chl-a peak at 3 m observed in spring (April) was maintained during the next months (July) with only a slight increase in intensity. A second peak appeared at around 5–6 m depth in July. This seasonality of Chl-a profiles either denotes the development of a summer bloom or the migration of a given population to deeper waters as light penetrates deeper in summer (Figure 5a,b). However, under low PAR conditions some organisms produce more chlorophyll [43], meaning that higher Chl-a concentrations may not be a reflection of algal abundance. In both cases (spring and summer), the position of the DCM was located near the bottom of the thermocline (Figure 5a), suggesting that the position of this layer may also exert some control on the position of the DCM.

### 3.4. Relation between DCM, MOM and Microbial Biomass

**Figure 6:**
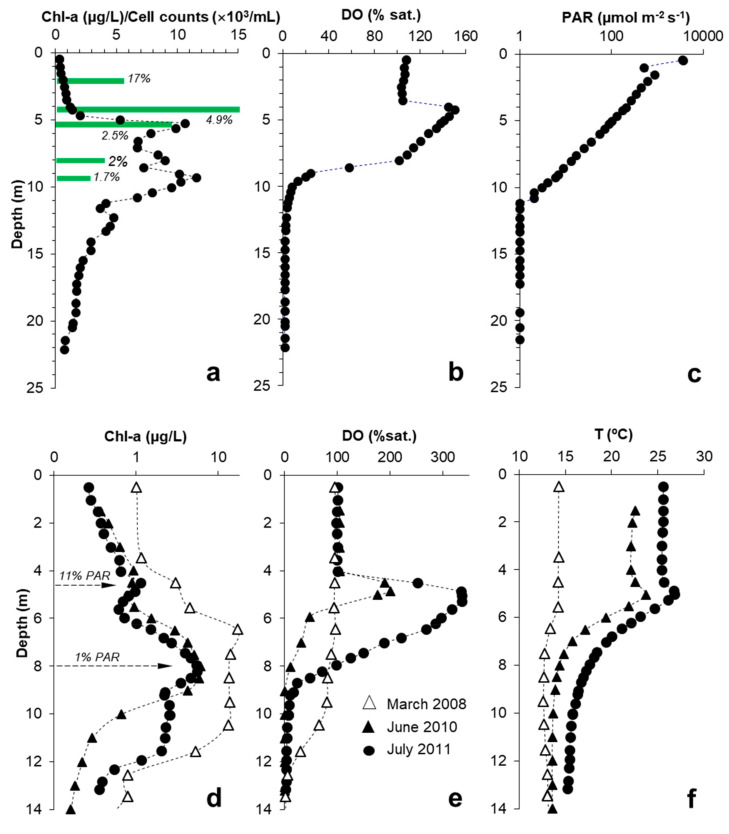
*Top:* Depth profiles of Chl-a concentration (**a**), DO concentration (**b**) and PAR (**c**) measured in July 2011 in Cueva de la Mora. The horizontal green bars in (a) indicate cell counts of phytoplankton measured at selected depths. The numbers in italics shown for each bar indicate the corresponding PAR intensity (with respect to that measured at the lake surface) in every case. *Bottom*: Depth profiles of Chl-a concentration (**d**), DO concentration (**e**) and temperature (**f**) obtained in three different seasons in the photic zone of Herrerías-Guadiana. The arrows in (d) indicate the PAR intensity (as % of that measured at the lake surface) in the two major Chl-a peaks.

In the biggest pit lake studied, ST, the photosynthetic activity has a moderate effect on the concentration of DO at depth, as illustrated in Figure 5c; the DO saturation level was around 70%-80% sat. in the upper four meters in April 2010 and approached 125% three months later. However, in the smaller pit lakes, CM or HER, the photosynthetic activity often produced marked MOM where DO concentrations drastically increased to values far exceeding saturation (Figure 6). This phenomenon occurs especially in summer, when a clear oxygen supersaturation is observed at some meters below the lake surface: in CM at 4.7 m the saturation of O_2_ was 150% in July 2011 (Figure 6b) and in HER was 200% to 340% O_2_ sat. at 5 m depth in June 2010 and July 2011, respectively (Figure 6e). These saturation values corresponded to DO concentrations around 12–13 mg/L O_2_ in CM and up to 22–24 mg/L O_2_ in HER. More recently, an outstanding MOM has also been observed in the Brunita (BRU) APL in July 2019 (320% O_2_ sat. at 2 m below the lake surface; see Figure S3). These oxygen concentrations are, to our knowledge, among the highest reported in lakes worldwide (either natural or artificial), and clearly reflect a strong disequilibrium between O_2_-producing (i.e., photosynthesis) and O_2_-consuming (e.g., aerobic bacterial metabolisms) processes at these depths.

The profiles in Figure 5 and Figure 6 clearly show that DCM, MOM and phytoplankton biomass (as deduced from cell counts) are not coincidental. In the case of ST in July 2011 (Figure 5a,b), the shallower Chl-a peak observed at 3.5 m (2.5 µg/L Chl-a) corresponded to a phytoplanktonic cell density of 382 cells/mL, whereas the deepest Chl-a peak developed at 5.8 m (10.6 µg/L Chl-a, which is four times higher than the concentration of the shallower peak) corresponded to a phytoplanktonic cell density of 3820 cells/mL (i.e., 10 times higher cell density than that of the shallower peak; Figure 5b). The deepest peak displaying the highest Chl-a concentration and cell density could not be related to any MOM (Figure 5c).

The MOM in CM and HER do not match with the corresponding DCM as seen in Figure 6. The Chl-a peaks are not related to maxima of biomass nor with higher photosynthetic activity; these DCM likely denote a higher production of Chl-a per cell due to the very low light conditions prevailing at those depths [5]. In CM (Figure 6a,b), the MOM observed at 4 m was likely caused by high density of chlorophyte cells (15,000 cells/mL), which were producing O_2_ at very high rates. However, the Chl-a concentration at that depth was relatively low (1.2 µg/L Chl-a). Conversely, the Chl-a peak observed at around 9 m with a higher Chl-a concentration (9.3 µg/L Chl-a; Figure 6a) corresponded with a layer of lower phytoplanktonic cell density (around 3000 cells/mL) and did not affect the DO concentration at that depth to a remarkable extent. In the case of HER, the MOM found at 4.5 m in June and July 2011 did match with the shallower Chl-a peak detected at the same depth (Figure 6d,e). In this case, the microalgae forming this shallow chlorophyll peak would have been also producing oxygen at much higher rates than the communities inhabiting deeper waters (8 m), despite their relatively lower chlorophyll content. Although no cell counting was conducted in this case, the MOM observed in HER was likely produced by a higher concentration of phytoplanktonic cells with average chlorophyll content, while the DCM would have been formed by a different population with lower density but higher chlorophyll content per cell. Although probably of minor importance, some contribution by other phototrophs containing other pigments (e.g., bacteriochlorophyll, carotenoids, phycoerythrin or phycobilins) cannot be completely ruled out.

**Figure 7:**
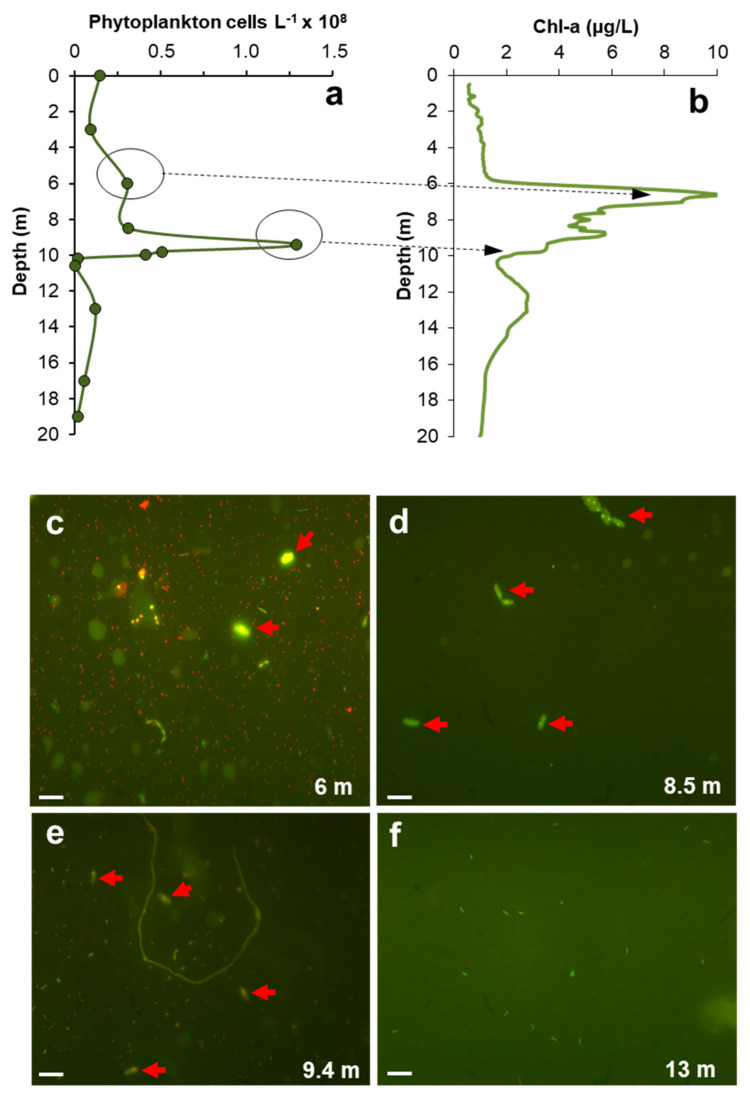
Vertical profiles of phytoplankton cell counts (**a**) and Chl-a concentration (**b**), and sequence of epifluorescence microscope images (**c**–**f**) showing the fluorescence intensity of phytoplanktonic cells (**c**–**e**; red arrows) and bacteria (**f**) at different depths in CM in September 2011. The scale bar in c–f represents 10 µm.

The comparison of phytoplanktonic cell counts measured in CM in September 2011 (Figure 7a) with the corresponding Chl-a profile (Figure 7b) clearly shows that the maximum of phytoplankton cell density at 9.4 m (1.25 × 10^8^ cells L^−1^) corresponded with a low Chl-a concentration of 3.5 µg/L. Conversely, the maximum content of Chl-a measured at a depth of 6 m (10 µg/L Chl-a) corresponded with a relatively low phytoplanktonic cell density of less than 3 × 10^7^ cells L^−1^ (Figure 7a,b). Epifluorescence microscope analysis on samples from different depths illustrate how the DCM found at 6 m was actually caused by sparse phytoplanktonic cells with a deeply bright green color suggestive of a high chlorophyll content (Figure 7c). On the other hand, the picture of the sample from 9.4 m (depth of maximum phytoplanktonic cell counts) showed cells with weaker fluorescence indicative of low Chl-a content, which is also coherent with the low Chl-a concentration measured at that depth (Figure 7e). At 13 m only bacterial cells were observed and phototrophs were absent in many microscopic fields (Figure 7f).

### 3.5. Thermal Effects of MOM

A striking but little studied feature related to the presence of MOM refers to the water temperature at depths where the MOM are developed. As illustrated in Figure 6e,f, marked water temperature peaks have been found in pit lakes like HER, which are fully coincidental with the DO peaks. This is not accidental, but a rather frequent situation in summertime when solar radiation is more intense. This phenomenon has also been observed in BRU, where an intense MOM observed in July 2019 (325% O_2_ sat. at a depth of 2 m) coincided with a marked increase of the water temperature (Figure S3 in Supplementary Materials). These thermal anomalies are outstanding and imply changes in water temperature between 1.5–2 °C (e.g., HER) and up to 4 °C (BRU) with respect to the temperature existing in the surrounding water. This phenomenon has been described and explained by mathematical modelling in ice-covered Arctic lakes in Canada [44] and in the ocean [45], but to our knowledge, these T anomalies at a depth related to the presence of phytoplankton had not been observed in temperate lakes. This situation is considered to result from a biophysical feedback effect where presence of pigmented microbes affects the local absorption of PAR, thereby causing an increased accumulation of heat in the surrounding waters [44]. Thus, much of the light absorbed by the phytoplanktonic communities existing in the MOM would not be used in photosynthesis but dissipated and released in the form of heat to the surrounding water.

### 3.6. Diurnal Phytoplankton and DO Dynamics

The vertical profiles of Chl-a and PAR obtained in the Barruecopardo pit lake (Figure 4c,d) suggests some diurnal dynamics of phytoplanktonic communities in response to changing light conditions. During a winter morning, the Chl-a maximum appeared to migrate downwards as the solar radiation became stronger and PAR reached deeper waters. This pit lake is also acidic (pH 3.5) but shows a much lower Fe(III) concentration (3.5 mg/L) with respect to other APL in the IPB (Table 2). The photic zone is deeper (i.e., 20–30 m) and Chl-a could be detected in the entire water column from surface to bottom. Chlorophyll-a increased with depth until reaching a maximum concentration that remained constant for the remaining of the water column. At 9:30 am, the Chl-a maximum was detected at 9–10 m; sometime later (at 10:45 am) a second peak started developing at 20 m, but after midday (12:40 pm) the shallower peak had moved down to 15 m and the deeper peak had moved down to 25 m (Figure 4d). The PAR intensities at 20 and 25 m were 3 and 2 µmol m^−2^ s^−1^ (0.3% and 0.1% PAR), respectively. Temperature and specific conductivity profiles (not shown) showed that the entire water column of the Barruecopardo pit lake was homogeneous, fully mixed, with no vertical density gradient driven by temperature or conductivity. Thus, this vertical evolution of Chl-a was not provoked by differential settling nor by nutritional needs and could be related to other processes such as photo-acclimation [5].

DO measured by loggers during three consecutive days at different depths in CM in September 2011 (Figure S4) showed certain diel cycles of DO concentration. During the three-day period, the concentrations measured at depths of 5.2 and 6.5 m (12 to 13 mg/L O_2_) corresponded with highly supersaturated conditions (around 140%–150% sat.) due to intense photosynthetic activity by the algae. However, the loggers detected sudden drops of DO concentration twice a day, at around 9–10 pm (UTC+2; sunset) and around 10–11 am (UTC+2) in the morning, whereas temperature dial fluctuations were in the range of ±1 °C (data not shown) and, thus no variation of oxygen solubility would be expected. In deeper waters (e.g., at 8 m and 9 m depth), no significant changes in DO concentration were detected (Figure S4). The observed cyclic decreases of DO concentration at 5.2 and 6.5 m are presently unknown, though they might have been caused by many different physical and ecological processes, including both O_2_-producing and O_2_-consuming processes that control O_2_ concentration at different time scales in lakes [46]. Among these processes, predation by other microorganisms feeding on phototrophic algae at certain hours could be a plausible possibility, however, the low abundance of other eukaryotes rather than phototrophs makes this hypothesis unlikely. Another possibility that should be considered is that part of the communities existing at these depths are motile and could migrate to lower waters as a strategy to balance between light and nutritional needs. Motile phytoplanktonic cells may move through the water column at speeds of some tens of cm per hour, and up to 90 cm per hour, depending on the species and on environmental conditions [42]. It could also be argued that the O_2_ drops do not represent a decline in photosynthetic O_2_ production but an increase in some O_2_-consuming processes, such as aerobic bacterial metabolisms. In any case, these diel cycles of O_2_ concentration remain unclear and will need further investigation.

### 3.7. Nutrient Availability

**Figure 8:**
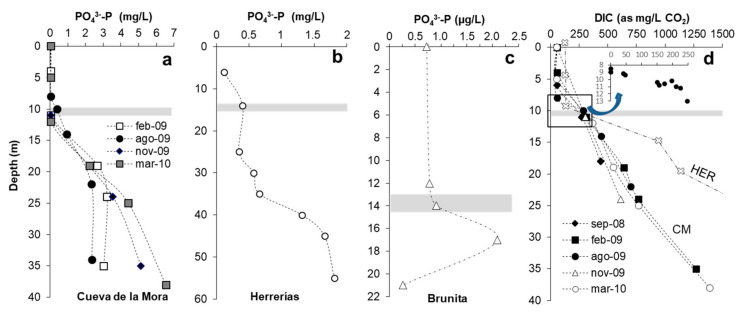
Vertical profiles of phosphate phosphorus concentration obtained in Cueva de la Mora (different seasons, **a**), Herrerías (July 2011, **b**) and Brunita (Nov 2018, **c**). (**d**) Vertical profile of dissolved inorganic carbon (DIC, as mg/L CO_2_) in Cueva de la Mora (CM) and Herrerías (HER) APL as measured in different seasons. The inset shows magnification of the chemocline zone with a high resolution profile conducted in September 2011 between 8 and 13 m depth in CM. The DIC concentration at depth in HER is much higher (5000 mg/L) and is not represented for clarity. The shaded area indicates the approximate position of the chemocline in all cases.

**Figure 9:**
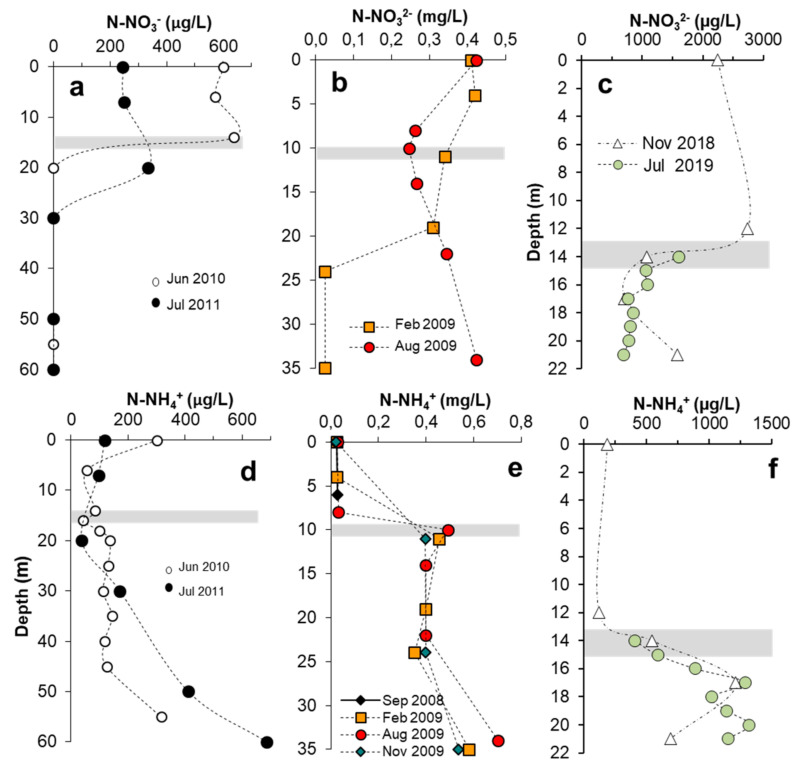
Vertical variation of nitrate-nitrogen (N-NO_3_^−^, **a**–**c**) and ammonium-nitrogen (N-NH_4_^+^, **d**–**f**) concentration in Herrerías (**a**,**d**), Cueva de la Mora (**b**,**e**) and Brunita (**c**,**f**) acidic pit lakes, as measured in different seasons. The shaded area indicates the approximate position of the chemocline in all cases.

Generally, AMD is considered to have very limited availability of basic nutrients like phosphate, nitrogen or inorganic carbon, and APL are usually known to be oligotrophic systems [16,17,18]. Analysis of the availability and vertical/seasonal distribution of these basic nutrients in APL is therefore essential to evaluate their influence on phytoplanktonic growth and distribution. Concentration of some of these nutrients measured in different pit lakes are provided in Figure 8 and Figure 9 and Table S2.

#### 3.7.1. Phosphorus

Phosphorus is usually the most critical nutrient, limiting algal growth in freshwater lakes [1] and also in acidic coal-mining lakes [16,17,18]. The profiles of Figure 8 show phosphate phosphorus (P-PO_4_^3−^) concentrations at depth in CM, HER and BRU measured between 2009 and 2019. In all cases, P-PO_4_^3−^ clearly increases with depth, and mixolimnetic concentrations are usually below detection (<25 µg/L P-PO_4_^3−^) in CM and between 30 and 65 µg/L P-PO_4_^3−^ in HER (Table S2, Figure 8), reaching higher concentrations below the chemocline (e.g., 390 µg/L P-PO_4_^3−^ at 10 m in CM and 413 µg/L P-PO_4_^3−^ at 14 m in HER) and at the bottom of the water column (between 2 mg/L and 6 mg/L). The scarcity of phosphate in the mixolimnion is mostly due to adsorption of this molecule to schwertmannite [25]. The absence of Fe(III) in the anoxic monimolimnion, the reduction of schwertmannite (and release of adsorbed phosphate) by iron reducing microorganisms and the continuous release of phosphate from the sediments (by desorption) and aluminosilicate dissolution present in host rocks provokes an increase of this nutrient in the monimolimnion. Phosphorus, therefore, is a likely factor forcing phytoplanktonic algae to move downwards to deeper waters. An exception has to be made in the particular case of BRU, where phosphate minerals exist in the open pit which are readily soluble at low pH. Phosphorus concentrations found in this pit lake in the mixolimnion (720–780 µg/L P-PO_4_^3−^) and below the chemocline (2100 µg/L P-PO_4_^3−^; Table S2) far exceed those found in the IPB pit lakes and in coal mine pit lakes of the Lusatia mining district (6–25 µg/L P_T_ [16,17,18]).

#### 3.7.2. Dissolved Inorganic Carbon

In contrast to the German APL in the Lusatia mining district (where CO_2_ is usually <0.1 mg/L and DIC is normally rather low), the APL of the IPB usually show very high concentrations of DIC (mostly CO_2_) as a result of carbonate dissolution during water/rock interaction (e.g., acid dissolution of calcite-containing volcanic and sedimentary rocks) [47]. Carbon dioxide increases sharply with depth in the monimolimnion (Figure 8d) in CM and HER, and although the concentrations in the mixolimnion are relatively lower (e.g., 40–83 mg/L CO_2_; Table S2, Figure 8d) it would be, a priori, sufficient to cover the phytoplanktonic nutritional needs. The sharp upwards decline of CO_2_ shown in the detailed profile of Figure 8d (inset with high-resolution sampling across the transitional chemocline of CM) suggests an intense consumption of CO_2_ in the zone where the deepest DCM are usually formed. This could be indicating that the proximity to the CO_2_-rich monimolimnion could represent a nutritional advantage for microalgal species well adapted to extremely low light intensities. Some species of green algae such as *Chlamydomonas reinhardtii* have been found to grow faster and produce more biomass (both photoautotrophically and mixotrophically) under CO_2_-rich conditions [48]. This would also be in agreement with a recent study in El Sancho reservoir that showed that the vertical distribution of CO_2_ during summer stratification is the main driver of DCM formation [19]. In any case, some physical factors like mixing and diffusion of CO_2_ through the chemocline must be also considered.

#### 3.7.3. Nitrogen

Inorganic nitrogen is also present at significant amounts in the APL. Nitrate nitrogen (N-NO_3_^−^) usually follows the opposite trend to that of ammonium nitrogen (N-NH_4_^+^) (Figure 9). Nitrate is relatively high in the oxygenic mixolimnion and decreases or virtually disappears with depth (except in the profile of August 2009 in CM); contrarily, ammonium is very scarce in the mixolimnion and increases in the anoxic monimolimnion. This opposed relation between nitrate and ammonium is also observed on a temporal basis (e.g., marked decrease of nitrate concentration at 10 m depth in CM in August 2009 paralleled by a corresponding increase of ammonium at the same depth; Figure 9b,e). These observations suggest biogeochemical cycling of nitrogen, though undemonstrated, since both nitrification and dissimilatory nitrate reduction to ammonium are thermodynamically unfavorable at the low pH conditions prevailing in these APL [49]. Considering both chemical forms (nitrate and ammonium), dissolved inorganic nitrogen (DIN) concentrations in the photic zone of the studied lakes are 350–900 µg/L in CM, 260–424 µg/L in HER, and 2500–2900 µg/L in BRU. The higher chlorophyll concentrations (DCM intensity) and phytoplankton diversity in CM with respect to HER and ST may be also related to higher DIN content in CM. The abundance of nitrate in the upper water layers suggests that N-NO_3_^−^ is probably not being utilized for algal growth in these pit lakes as they usually prefer ammonium as a source of N rather than nitrate as it is easier to assimilate [50]. Thus, ammonium nitrogen could also be a potential factor driving the formation of DCM in APL like CM.

#### 3.7.4. Silica

Although silica is not a basic nutrient for green algae, it has been included in Table S2 as it is an important constituent for diatoms, which have been found in water column samples of the studied APL (Figures S5–S6). Dissolved silica is abundant at all sampling depths in all pit lakes, and concentrations (79–116 mg/L SiO_2_) are sufficient in the entire photic zone to ensure frustule accretion, so it is very unlikely that it plays any role in DCM formation.

### 3.8. Phytoplankton Community Composition

A revision of the phototrophic microorganisms identified so far at different depths and by different techniques in APL of the IPB is provided in Table 3. Microscopic examination of samples collected at the DCM of CM, HER and ST pit lakes revealed cells of flagellated phototrophs, probably Chlamydomonadales, diatoms and other non-phototrophic eukaryotes such as ciliates or heliozoans [51] (Figure S5 in Supplementary Materials). A deeper microscopic examination of the phytoplankton of surface waters in CM [22] revealed the presence of unicellular and flagellated green algae of the *Chlamydomonas* genus, in addition to other phototrophs like filamentous green algae of the genus *Zygnema*, diatoms with different morphology, and unicellular flagellated cells of *Ochromonas*. *Ochromonas* is a predatory mixotroph that can combine photosynthesis with prey uptake at the expense of *Chlamydomonas* [16,17,18].

Later studies of the eukaryotic communities in deeper waters (i.e., near the DCM and/or MOM) by Terminal Restriction Fragment Length Polymorphism (T-RFLP) [25,51] revealed the presence of other phototrophic microorganisms (Table 3). In these studies, two acidophilic phototrophs, *Coccomyxa* sp. strain AC1 and *Auxenochlorella protothecoides var. acidicola* (strain CFR26), were isolated on solid media from water collected at 3 m and 6 m, respectively, from the CM pit lake [25]. The dominant T-RFLP found in the DCM of HER pit lake corresponded to *Coccomyxa* sp. strain AH4 that was identical to *Coccomyxa* sp. strain AC1. Later work revealed that both strains AC1 and AH4 correspond to *Coccomyxa* (*Cc.*) *onubensis* (Trebouxiophyceae, Chlorophyta; [52]). The T-RFLP profiles of the epilimnetic waters of HER presented similar eukaryotic community in samples taken in March 2012 and in September 2012, whilst CM showed variations of the phototrophic community between these two sampling periods [25,51]. *Coccomyxa onubensis* has been recently found in the nearby Tinto river at pH ~2.5 [52]. This phototroph yields maximum biomass productivities at pH 2.5–4.5 and tolerates salinities up to 0.5 M NaCl, so that this microorganism is both acido- and halotolerant [53]. Besides showing a robust response to hyperosmotic shock, this phototroph can adopt very different morphologies depending on salinity [53], a circumstance that has probably led to confound this microorganism with other species. Recent studies have shown that *Coccomyxa* spp. is able to grow in a wide range of habitats including metal-contaminated streams in Sardinia or APL in the Czech Republic [54,55]. Members of genus *Coccomyxa* spp. may also grow mixotrophically, which further extends their habitat range [56]. Its flexibility and adaptability gives *Coccomyxa* spp. a clear advantage under extreme conditions and may become the dominant alga, overriding other phototrophs like *C. acidophila* which usually succeed in similar systems.

**Figure 10:**
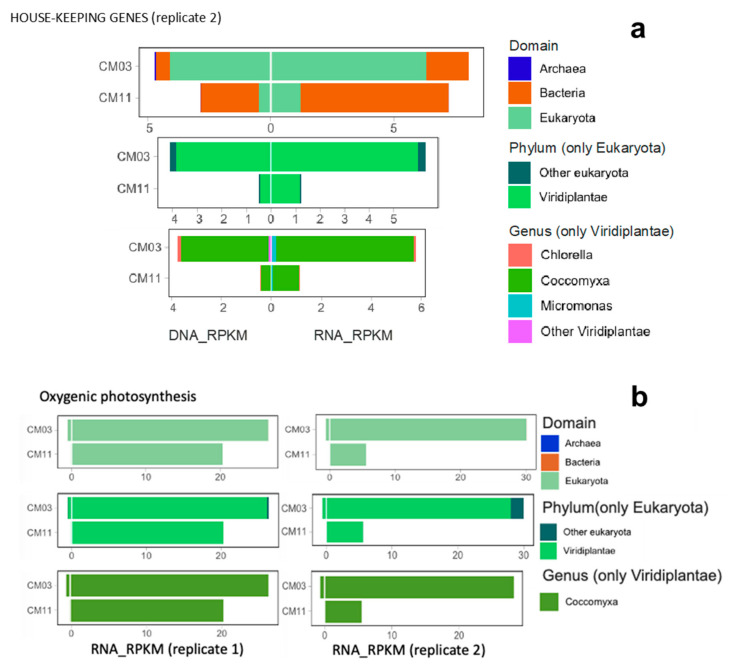
(**a**) Total frequency (DNA_RPKM) and expression (RNA_RPKM) of predicted genes functionally annotated as house-keeping genes obtained from metagenomic and metatranscriptomic analysis conducted in the APL Cueva de la Mora. RPKM refers to Reads mapped to a predicted gene per Kilobase (length of the predicted gene) per Million Reads (total number of reads mapped to all predicted genes found in the metagenome or metatranscriptome): (top) barplot representing the total sum of RPKM values for the predicted genes affiliated to the three different domains; (center) same as (top) with the bars broken down by phylum only focused on Eukaryota; (bottom) total sum of RPKM values for the predicted genes affiliated to specifically the phylum Viridiplantae (green algae) at the genus level. Note: replicate 1 (given in Figure S7) showed exactly the same results. (**b**) Total frequency expression (RNA_RPKM) of predicted genes functionally annotated as part of oxygenic photosynthesis obtained from metatranscriptomic: (top) bar plot representing the total sum of RPKM values for the predicted genes affiliated to the three different domains; (center) same as (top) with the bars broken down by phylum only focused on Eukaryota; (bottom) total sum of RPKM values for the predicted genes affiliated to specifically the phylum Viridiplantae (green algae) at the genus level.

New results obtained in this study by the combination of shotgun metagenomics and metatranscriptomics have confirmed the dominance of *Coccomyxa* spp. in waters collected at 3 m (MOM) and 11 m (chemocline) in CM (May 2018). Both samples revealed nearly identical phototrophic communities, both in relation to the abundance (DNA_RPKM) and the expression (RNA_RPKM) of genes (Table 3; Figure 10a). This meta-omics approach points to a very low diversity, with dominance of *Coccomyxa* sp. among the eukaryotic organisms (Figure 10a). The sample taken at 3 m corresponded to a clear MOM developed at the time of sampling (160% sat. DO, not shown), therefore, *Coccomyxa* sp. (mostly likely *Cc. onubensis*, as found in earlier studies) can be considered the microorganism causing the formation of the MOM. Interestingly, the expressed genes detected at 11 m (i.e., below the chemocline, with extremely low PAR of ~1 µE m^−2^ s^−1^ and with toxic concentrations of bacterial H_2_S [24,25]) indicates that *Coccomyxa* sp. was not only present but also active, meaning that this microalga is able to thrive at that depth and probably obtains some ecological benefit related to nutrient uptake, as discussed below.

The results reveal that the phytoplankton community of CM has a low diversity where *Coccomyxa* sp. dominates above all other genera. The combination of gene abundance and gene expression indicates that green algae in general, and *Coccomyxa* sp. in particular, are the most important O_2_ producers at the sampled depths (3 m and 11 m), representing >95% of the oxygenic photosynthesis in this pit lake (Figure 10b). The remaining 5% of the oxygen production would be conducted by other phototrophs. Among the latter, evidence of the presence of C*yanidiophyceae* has been found. Laboratory cultures conducted in 2017 with samples taken at different depths (5, 6 and 7 m) in CM pit lake revealed the presence of red algae (Figure S6 in Supplementary Materials). These cultures were grown at two different pH conditions (1.5 and 3.5) to cover the spectrum of pH observed in pit lakes of the IPB [20]. In the cultures grown at higher pH (3.5), an unknown diatom species grew in the sample from 5 m depth, while cells very similar to *Cc. onubensis* grew in the deeper sample from 7 m (Figure S6). However, in the cultures grown at pH 1.5, the phototroph which proliferated most conspicuously at all depths was an organism with blue-green coloration characteristic of certain red algae (Figure S6). An isolate whose chloroplast DNA was related to *Cyanidium caldarium* was isolated from the sample collected at 5 m, though further confirmation on species identity was not carried out. The presence of cyanidiales in these waters would represent novelty because they are a small family of asexual, unicellular, thermoacidophilic red algae usually found in very acidic (pH 0.5–3.0) and hot (50–55 °C) geothermal systems [57,58,59,60].

### 3.9. General Considerations on the Phototrophic Communities and Their Role in DCM and MOM Development

The composition of the phototrophic community of the studied pit lakes is rather different with respect to those described in other APL in Germany and Spain and in other acidic waters of the IPB (Table S3). For example, in ML 111, an extensively studied APL in Lusatia mining district (Germany), the green microalga, *C. acidophila,* was found to be the main responsible for the formation of DCM [16,17,18]. *Chlamydomonas acidophila* is well adapted to extremely low light conditions and can colonize deeper waters where nutrient availability is higher and predatory pressure is low [18]. Its high tolerance to low pH (optimal growth at pH 2.5–3.5, but seen to grow at pH as low as 1.5) and high metal concentrations (e.g., Zn, Cd, Cu, Co) makes this microorganism ubiquitous in acidic lakes and ponds [61]. It is also a very common species in acidic volcanic lakes [62,63]. Another genus of the *Chlamydomonadaceae* family, *Carteria* sp., was found to be dominant in the DCM (19–20 m) of the AMD-impacted water reservoir El Sancho (SW Spain), which is also acidic (pH 3.5–4.0) but exhibits much more dilute and less toxic chemical conditions than the APL of this region [19]. The genus *Ochromonas* spp. is also rather abundant in many German pit lakes along with *Euglena mutabilis* and some diatom species, which usually form dense mats on the littoral sediments [64]. *Chlamydomonas* spp. has been also found to be an abundant phytoplankton component in at least two other APL in SW Spain, Concepción and Nuestra Señora del Carmen [65]. In Concepción, *Chlamydomonas* sp., *Ochromonas* sp. and other unidentified eukaryotic phototrophs were detected, whilst in Nuestra Señora del Carmen only *Chlamydomonas* spp. was detected and only from the surface water sample [65]. The phototrophic community of the studied pit lakes is also notably different to those found in acidic streams of the area (e.g., Tinto, Tintillo and Perrunal rivers, Cantareras stream), where the chlorophytes *Euglena* spp., *Dunaliella* spp, *Chlamydomonas* spp., *Chlorella* spp. and *Zygnema* spp., in addition to different diatom species, are usually the dominant benthic algae [66,67,68,69,70].

*Coccomyxa* sp. has been recently reported to be the major phytoplankton component in Hromnice Lake in the Czech Republic [55]. This APL has a very similar chemical composition to those found in CM and HER, including a low pH (~2.6), very high sulfate and metal (Fe, Al, Mn, Ni, Cu, Co) concentrations, and relatively high phosphate concentrations, which also increase with depth [55]. These data demonstrate that *Coccomyxa* spp., a highly opportunistic microorganism, is able to colonize very acidic and metal-rich environments. The phytoplanktonic phototrophs observed in the DCM and MOM of CM and HER, especially *Coccomyxa* spp., are not only adapted to severe acidity (pH close to 2.0 depending on the season; Table 2), very high salinity (5–10‰) and high concentrations of many toxic metals (Table S1), but they must also cope with extremely low light conditions. These characteristics make *Coccomyxa* spp. a poly-extremophile.

Regarding the presence of *Auxenochlorella* spp. in the studied APL, the occurrence of this phototroph in low pH environments was previously thought to be restricted to acidic soils [71]. However, this genus has been already found as the dominant benthic algae in very acidic and metal-rich streams of the area like the Tinto river [66,67] or the Perrunal creek [69]. *Auxenochlorella protothecoides var. acidicola* is morphologically identical to *A. protothecoides*; the only diacritical character for separating this variety is a pH limit for growth at 2.0 compared to 3.5–4.0 for *A. protothecoides* [72]. In addition, *A. protothecoides var. acidicola* has been shown to grow at salinities of up to 3–5‰ and temperatures up to 34 °C; this species is apparently not able to grow on nitrate [72].

With respect to detection of cyanidiales, these algae are considered to be some of the most ancient algae in the eukaryotic evolution and they are extremely simple, with just one chloroplast and one mitochondrium. Most of these algae are in fact blue–green due to the pigments c-phycocyanin and chlorophyll-a [73] and also present a variety of pigments, including betacarotene and zeaxanthin [73]. *Cyanidium caldarium* is the most studied member among the cyanidiales, being characterized by rounded cells with thick walls, which apparently help to cope with extreme acidity (pH close to 0) and temperatures up to 55 °C [57,58,59,60]. A new strain within the *Cyanidium* genus has been recently found in caves with virtual absence of light [74]. Thus, the finding of *Cyanidium* spp. at depths of 6 and 7 m in CM, where PAR intensity is well below 1% of that at the lake surface (Figure 4a,b), is not surprising. To the best of our knowledge, this is the first time that cyanidiales are reported in APL. These ancient and rather simple species of eukaryotic phototrophs have been able to colonize some of the most extreme habitats on Earth thanks to their extremely high adaptive and resistant capacity [57,58,59,60,74]. Our results suggest that the cyanidiales are present in very low numbers in these APL, being almost undetectable by T-RFLP and meta-omics approaches, but they could proliferate in the case of further acidification of these systems in the future.

Among the mechanisms proposed to explain the formation of DCM in lakes worldwide (Table 1), those of differential sinking of non-motile epilimnetic algae (e.g., diatoms, mechanism 3 [9,10]) or photo-inhibition in near-surface waters due to high UV radiation exposure (mechanism 6 [11]) are apparently unimportant in the studied pit lakes. Regarding the differential sinking, observations obtained in HER and also in other pit lakes of IPB like Confesionarios or Concepción [21] suggest that a sharp thermocline or density gradient is actually not required for the development of DCM. This mechanism could only be relevant in the formation of the deeper Chl-a concentrations found below the chemocline (e.g., at 12–13 m in CM; Figure 6 and Figure 7) since these deeper peaks are apparently caused by the accumulation of senescent algal cells rich in phaeophytin (Figure 3). With respect to photo-inhibition, previous studies have shown that UV radiation is strongly attenuated in the first centimeters of the water column [26]. Thus, with the exception of Barruecopardo (with very low Fe(III) content that allows UV light reach deeper levels and may lead to photoinhibition; Figure 4d), this factor is likely not a mechanism contributing to the formation of DCM in most acidic pit lakes.

The presence of ciliates and other protozoans in the studied pit lakes, coupled with the detection of *Auxenochlorella* spp., would a priori allow mechanism 4 (i.e., symbiosis between microalgae and protozoa [11]) to some extent, though the low concentration of non-photosynthetic eukaryotes by meta-omics analyses suggests that this mechanism is highly unlikely. Based on the data presented in this study, we propose that the combination of mechanisms 1 (in situ growth of *Coccomyxa* sp. and other phototrophs promoted by the higher nutrient availability below the chemocline, including phosphorus, nitrogen and CO_2_ [8]) and 5 (photo-acclimation of phytoplankton by increased chlorophyll content per cell [12,13]) can probably explain the formation of DCM in the studied pit lakes. The need for nutrient uptake in oligotrophic systems has been widely proposed as one important mechanism driving the formation of DCM in stratified lakes [8,16,17,18] (Table S3). The scarcity of phosphorus, nitrogen or inorganic carbon for basic metabolic functions provokes the need for phototrophs to reach deeper waters (i.e., close to the upper part of the monimolimnion) where the limiting nutrient(s) are present at higher concentrations [8].

The lack of coincidence between DCM and MOM in the IPB pit lakes suggests that the deep chlorophyll-rich layers are actually formed by a relatively low density (low biomass) community dominated by *Coccomyxa*, which has apparently developed higher Chl-a content per cell to increase the photosynthetic yield at extremely low light conditions. This inverse relation between chlorophyll fluorescence signal and phytoplanktonic cell density, which is well illustrated in Figure 5, Figure 6 and Figure 7, is somehow counterintuitive and contradicts the general assumption that DCM represent peaks of phytoplanktonic biomass. The phototrophs identified in CM and HER (e.g., *Coccomyxa* spp.*, Auxenochlorella* spp.*, Chlamydomonas* spp.) are able to produce more chlorophyll to make photosynthesis still viable at light intensities as low as 0.1 µmol s^−1^ m^−2^. However, the red light spectrum is more difficult to use for photosynthesis and requires the production of other pigments such as phycoerythrins, phycocyanin or phycobilins [5]. These pigments are usually found in rhodophytes, so the finding of cyanidiales in CM at 5 m suggests that these microorganisms could be also contributing to the formation of DCM.

## 4. Concluding Remarks

The phototrophic community detected in CM (used here as a model system representing a typical case of meromictic, acidic and metal-rich pit lake) has a low diversity dominated by *Coccomyxa* spp., a highly versatile genus. *Coccomyxa* spp. is known to simultaneously resist different environmental stressors and carry out photosynthesis at extremely low light conditions. The formation of DCM in the studied pit lakes is mostly the result of the colonization of deeper waters (e.g., 11 m) by *Coccomyxa* spp., which can probably utilize higher nutrient resources, including phosphate phosphorus, ammonium nitrogen and carbon dioxide, which diffuse through the chemocline from the upper monimolimnion to the lower mixolimnion. An obligate adaptation mechanism for taking advantage of these nutrients is likely the production of higher chlorophyll concentration per cell to cope with extremely low photon fluxes (even below 1 µmol m^−2^ s^−1^). At these low light conditions, however, *Coccomyxa* spp. appear to maintain a reduced photosynthetic activity, so that in most cases these DCM do not represent hot spots of oxygen and primary carbon production. In shallower waters (e.g., 3–5 m depth), PAR is higher (e.g., 10–100 µmol m^−2^ s^−1^, representing around 3–4% of that at the lake surface) and conditions are more favorable for the proliferation of *Coccomyxa* spp. and other microorganisms like *Auxenochlorella* spp. The higher light availability in shallower waters leads to higher biomass accumulation and cell densities and higher rates of oxygen production per volume unit, resulting in the formation of striking MOM and temperature anomalies in pit lakes such as CM or HER. The deeper chlorophyll peaks found below the chemocline (e.g., at depths of 12–13 m) are mostly produced by the accumulation of senescent cells settling from upper waters, as evidenced by the abundance of phaeophytin and virtual absence of viable cells of phototrophic microorganisms. However, the detection of active genes expressed at 11 m in CM suggests the possibility of *Coccomyxa* spp. being still viable below the chemocline in anoxic waters.

The physico-chemical characteristics of APL shape the phototrophic communities to a great extent. However, the nature and ecological interrelations of phototrophs and other eukaryotes inhabiting these systems is not yet well established. The development of a large phytoplanktonic community may be used to remediate acidic waters as they supply organic carbon to heterotrophic microorganisms (e.g., sulfate reducers) and, thus, mobilize toxic elements. Understanding the dynamics of phototrophs in the acidic waters is therefore of great importance as this group of microorganisms have a great impact on biogeochemical cycles in these extreme environments.

## Figures and Tables

**Table 1:** Summary of mechanisms proposed for deep chlorophyll maxima (DCM) formation in lakes worldwide (taken from [5]).

Mechanism	Description	Examples	Refs.
1	In situ growth of phototrophs promoted by higher nutrient availability in metalimnion or upper hypolimnion	DCM formed by cryptophytes or cyanobacteria	[8]
2	Differential predation pressure between near-surface and deep layers	Grazing of *Cryptomonas* by zooplankton; grazing of *Clamydomonas* by *Ochromonas*	[5]
3	Depth-differential sinking of eplimnetic algae (passive or active)	DCM formed by non-motile phytoplankton (e.g., diatoms)	[9,10]
4	Symbiotic association of algae with protozoa followed by blooming in metalimnion	Symbiosis between *Chlorella* and ciliated protozoa	[11]
5	Photo-acclimation of phytoplankton by increased chlorophyll content per cell	Photoadaptation of *Dunaliella tertiolecta*; lakes with clear waters	[12,13]
6	Photo-inhibition in near-surface waters due to high UV radiation exposure	Alpine lakes with very clear waters	[11]

**Table 2:** Selected geographic (location), morphometric (area, depth, volume), and chemical (pH, Fe(III) concentration) features of the acidic pit lakes (APL) studied in this work (CM, HER, ST, BRU, BP) plus three other APL that are also plotted in Figure 1 (NS, FC, CF). Compiled from [20,21,22,23,24,25,26,27,28].

Lake	Location	Area	Depth	Volume	pH	Fe(III)
		m^2^	m	m^3^		mg/L
CM	IPB, Huelva	17,900	40	3 × 10^5^	2.3–3.2*	108
HER	IPB, Huelva	19,000	70	3 × 10^5^	2.0–3.1*	140
ST	IPB, Huelva	145,500	130	7 × 10^6^	2.2–3.0*	202
BRU	La Unión, Murcia	45,000	21	9.5 × 10^5^	2.0–2.4	450
BP	Salamanca	38,000	40	5 × 10^5^	3.5	2.4
NS	IPB, Huelva	7000	32	1 × 10^5^	2.5–2.8	330
FC	IPB, Huelva	38,000	50	6 × 10^5^	2.0–2.4	650
CF	IPB, Huelva	24,800	80	1 × 10^5^	2.3–2.5	812

*, pH values refer to the epilimnion and photic zone of the lakes. Abbreviations: CM, Cueva de la Mora; ST, San Telmo; HER, Herrerías; BRU, Brunita; BP, Barruecopardo; NS, Nuestra Señora del Carmen; FC, Filón Centro (Tharsis); CF, Confesionarios.

**Table 3:** Summary of phototrophic microorganisms identified at different depths and by different techniques in acidic pit lakes of the Iberian Pyrite Belt (IPB) (names in bold denote dominant microorganisms).

Lake	Depth	Date	Microorganisms	Technique	Source
	(m)				
CM	0	July 2007	*Chlamydomonas*, Zygnematales, *Ochromonas*, Diatoms	Confocal microscopy	[22]
CM	3	Sept 2011, March 2012, May 2018	*Coccomyxa sp., Chlorella, Micromonas,*	T-RFLP, Confocal, Microscopy, SMA	[25,51] *This study*
CM	5	Sept 2017	*Cyanidium caldarium,* Diatoms	Cultures, confocal microscopy	*This study*
CM	6	Sept 2012, Sept 2017	*Coccomyxa onubensis,Auxenochlorella*, *Cyanidium caldarium*	T-RFLP, Cultures, confocal microscopy	[25,51] *This study*
CM	7	Sept 2017	*Cyanidium caldarium*	Cultures and confocal microscopy	*This study*
CM	8	Sept 2011	*Coccomyxa onubensis,Auxenochlorella*, Diatoms	T-RFLP, Confocal microscopy	[25,51]
CM	11	May 2018	*Coccomyxa sp., Chlorella, Micromonas*	SMA	*This study*
HER	5	March 2012	*Coccomyxa onubensis,Chlamydomonas*, Diatoms	T-RFLP, Confocal microscopy	[25,51]
HER	7	March 2012, Sept 2012	*Coccomyxa onubensis,Chlamydomonas*, Diatoms	T-RFLP, Confocal microscopy	[25,51]

SMA, Shotgun metagenomic analyses. CM, Cueva de la Mora; HER, Herrerías.

## Supplementary Materials

The following are available online at https://www.mdpi.com/2076-2607/8/8/1218/s1, Table S1: Trace metal concentrations measured in the mixolimnion of the studied pit lakes, Table S2: Nutrient concentration measured at different depths in the studied pit lakes, Table S3: Revision of studies reporting formation of DCM in different lakes of the world, Figure S1: Geographic location and satellite images of the studied pit lakes, Figure S2: Details of filters used to sample water from the chlorophyll-rich levels in Cueva de la Mora pit lake, Figure S3: Vertical profiles of dissolved oxygen and temperature in the extremely acidic, metal-rich pit lake of Brunita mine, Figure S4: Diel cycles of O_2_ concentration as measured with dataloggers installed at different depths in Cueva de la Mora, Figure S5: Microscopic images of eukaryotic microorganisms observed at different depths in Cueva de la Mora, Herrerías and San Telmo, Figure S6: Microscope images of phototrophic microorganisms grown in cultures at room temperature with samples taken from Cueva de la Mora in September 2017, Figure S7: Replicate of Figure 10 with total frequency and expression of predicted genes functionally annotated as house-keeping genes obtained from metagenomic and metatranscriptomic analysis conducted in the APL Cueva de la Mora.
