# Supplementary material for: Adaptation of Coccomyxa sp. to Extremely Low Light Conditions Causes Deep Chlorophyll and Oxygen Maxima in Acidic Pit Lakes
Source: Microorganisms. 2020 Aug 11;8(8):1218. doi: 10.3390/microorganisms8081218 (PMC7465793; doi:10.3390/microorganisms8081218)
Supplement: Supplementary file 1 [file microorganisms-08-01218-s001.pdf]

Adaptation of *Coccomyxa* sp. to extremely low light conditions causes deep chlorophyll and oxygen maxima in acidic pit lakes

Javier Sánchez-España [1*], Carmen Falagán [2], Diana Ayala [3], Katrin Wendt-Potthoff [4]

1 Spanish Geological Survey, Calera 1, 28760 Tres Cantos, Madrid, Spain; j.sanchez@igme.es
2 University of Exeter, Penryn Campus, Cornwall, TR10 9FE, UK; c.falagan@exeter.ac.uk
3 The Pennsylvania State University, University Park, PA, USA; dka9@psu.edu
4 Helmholtz Centre for Environmental Research – UFZ, Magdeburg, Germany; Katrin.wendt-potthoff@ufz.de

Electronic Supplementary Material

Contains three tables (S1-S3) and seven figures (S1-S7)

Table S1. Trace metal concentrations measured in the mixolimnion of the acidic mine pit lakes studied in this work (compiled from [20-28]).

**Lake**	**Al**	**Cu**	**Zn**	**Mn**	**Co**	**Ni**	**Cd**	**As**	**Cr**	**Pb**
	mg/L	*mg/L*	*mg/L*	*mg/L*	*µg/L*	*µg/L*	*µg/L*	*µg/L*	*µg/L*	*µg/L*
CM	140	6	16	20	920	570	15	200	25	92
HER	76	21	130	158	3300	2500	186	44	34	26
ST	120	17	65	31	715	351	157	55	15	58
BRU	72	254	230	466	656	1040	195	40	31	59
BP	8	0.2	0.8	1.2	36	20	1.4	12	2	2

Abbreviations: CM, Cueva de la Mora; HER, Herrerías; ST, San Telmo; BRU, Brunita; BP, Barruecopardo.

Table S2. Concentration of dissolved inorganic carbon (DIC), phosphate phosphorus (PO_4^{3-}-P), total nitrogen (N_T), nitrate nitrogen (NO^{3-}-N), ammonium nitrogen (NH^{4+}-N) and soluble silica (SiO_2) measured at different depths in pit lakes of the Iberian Pyrite Belt.

**Pit lake**	**Layer type**	**Depth**	**Date**	**DIC**	**PO_4^{3-}-P**	**NO^{3-}-N**	**NH_4^+-N**	**SiO_2**
Units		m		mg/L	µg/L	µg/L	µg/L	mg/L
Cueva de la Mora	Mixolimnion, oxygenic	0	Feb 2009	57	b.d.	410	24	116
	Mixolimnion, oxygenic	4	Feb 2009	56	b.d.	420	25	116
	Mixolimnion, oxygenic	11	Feb 2009	310	b.d.	340	455	128
	Monimolimnion, anoxic	19	Feb 2009	640	2676	310	399	121
	Monimolimnion, anoxic	24	Feb 2009	768	3210	25	352	112
	Monimolimnion, anoxic	35	Feb 2009	1270	3030	25	580	80
Herrerías-Guadiana	Mixolimnion, oxygenic	0	June 2010	71	30	601	53	21
	Mixolimnion, oxygenic	6	June 2010	83	65	572	56	79
	Mixolimnion, oxygenic	7	Sept 2011	n.a.	30	250	96	n.a.
	Mixolimnion, oxygenic	14	June 2010	67	403	637	84	140
	Monimolimnion, anoxic	20	June 2010	1100	b.d.	b.d.	137	71
	Monimolimnion, anoxic	40	June 2010	2600	1310	b.d.	118	101
	Monimolimnion, anoxic	55	June 2010	4962	1810	b.d.	317	20
San Telmo	Mixolimnion, oxygenic	0	Sept 2008	40	31	n.a.	27	n.a.
	Mixolimnion, oxygenic	10	Feb 2009	48	b.d.	16	30	70
	Mixolimnion, oxygenic	20	Sept 2008	45	n.a.	n.a.	31	n.a.
	Monimolimnion, anoxic	40	Sept 2008	88	42	n.a.	47	n.a.
	Monimolimnion, anoxic	95	Feb 2009	104	b.d.	16	40	69

Table S3. Revision of studies reporting formation of DCM in different lakes of the world. n.r. not reported.

Lake	Location	Depth DCM (m)	PAR µmol m^{-2}s^{-1}	PAR (%I_0)	Dominant species	Mechanism proposed	Other factors	Ref.
Acidic lakes								
CM	IPB	4-9	8-15	0.2-0.4	*Coccomyxa* sp.	Nutrient uptake (P-PO_4^{3-}, N-NH_4, CO_2)	Photoacclim.	This study
ST	IPB, Spain	3-6	1-12	0.03-1	n.r.	Nutrient uptake (P-PO_4^{3-}, N-NH_4, CO_2)	Photoacclim.	This study
HER	IPB, Spain	6-10	6-100	0.2-2	*Coccomyxa* sp.	Nutrient uptake (P-PO_4^{3-}, N-NH_4, CO_2)	Photoacclim.	This study
BP	IPB, Spain	10-25	2-3	0.2-0.3	*n.r.*	Photoinhibition	*n.r.*	This study
El Sancho	IPB, Spain	22	<8	*n.r.*	*Carteria* sp. (Chlorophyta)	Nutrient uptake (CO_2)	Light	[19]
ML 111	Lusatia, Germany	6-7	3.1	0.2	*Chlamydomonas* sp.	Grazing by mixotrophic microorganisms (Ochromonas sp.) at upper levels	Light, CO_2	[16-18]
Caviahue	Volcanic lake, Patagonia, Argentina	30	*n.r.*	*n.r.*	*Keratococcus rhaphidioides* (chlorophyte)	Nutrient uptake (N-NH_4, P-PO_4)	DOC, pH, zooplankton biomass	[14]
Neutral to alkaline lakes								
Okaro	Volcanic lake, New Zealand	6-9	*n.r.*	1	dinoflagellates (*C. hirundinella*)	Nutrient uptake (N-NH_4)	Light, P-PO_4, predation	[11]
Cross	Reservoir, Kansas, USA	6-10	0.2-7.4	0.7	*Cryptomonas* (phytoflagellate)	Photoinhibition	Nutrient uptake	[9]
L20,L26, L39, L42	Boreal Great Lakes, Ontario, Canada	7-17	*n.r.*	≥1	chrysophytes, cryptophytes, diatoms	Photoinhibition	DOC	[12]
Rot	Mountain lake, Switzerland	4-8	0.02-1.2	0.01	Cyanophyceae, Bacillariophyceae	Photoinhibition	Nutrient uptake (N-NO_3)	[11]
Shira Shunet	Saline lakes, Siberia (Russia)	8-12	<0.4-2		*Dictyosphaerium* (green alga) *Lyngbya contorta* (cyanobacterium)	Differential sedimentation rates of algal biomass	Predation by zooplankton in upper levels	[10]
La Cruz	Cuenca, Spain	11	*n.r.*	0,1-1	Cyanobacteria Cryptophytes	Decrease in epilimnetic concentration due to grazing pressure	Nutrient (nitrogen) depletion in epilimnion	[5]

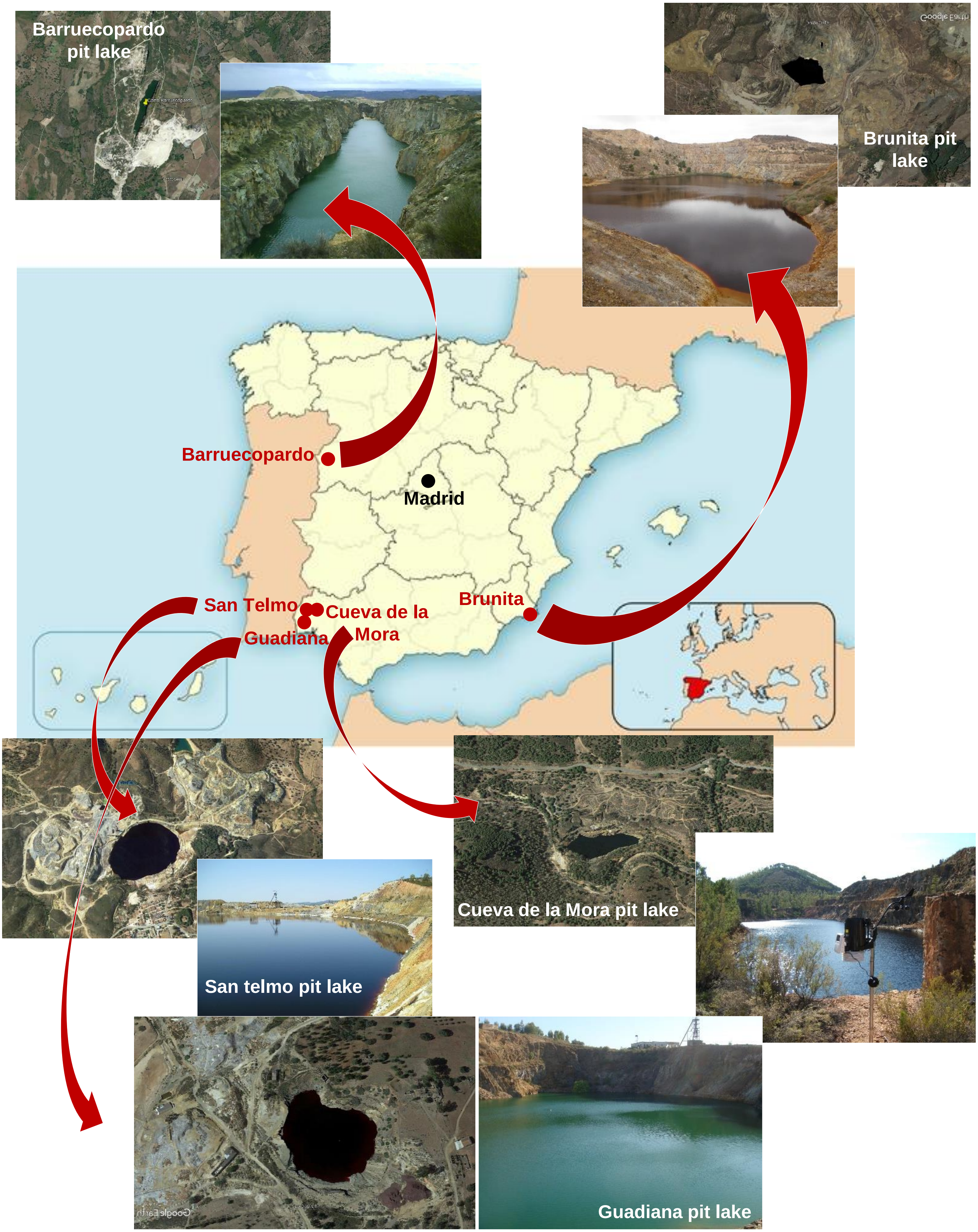

Figure S1. Geographic location, satellite images (Google Earth) and panoramic views of the studied acidic pit lakes.

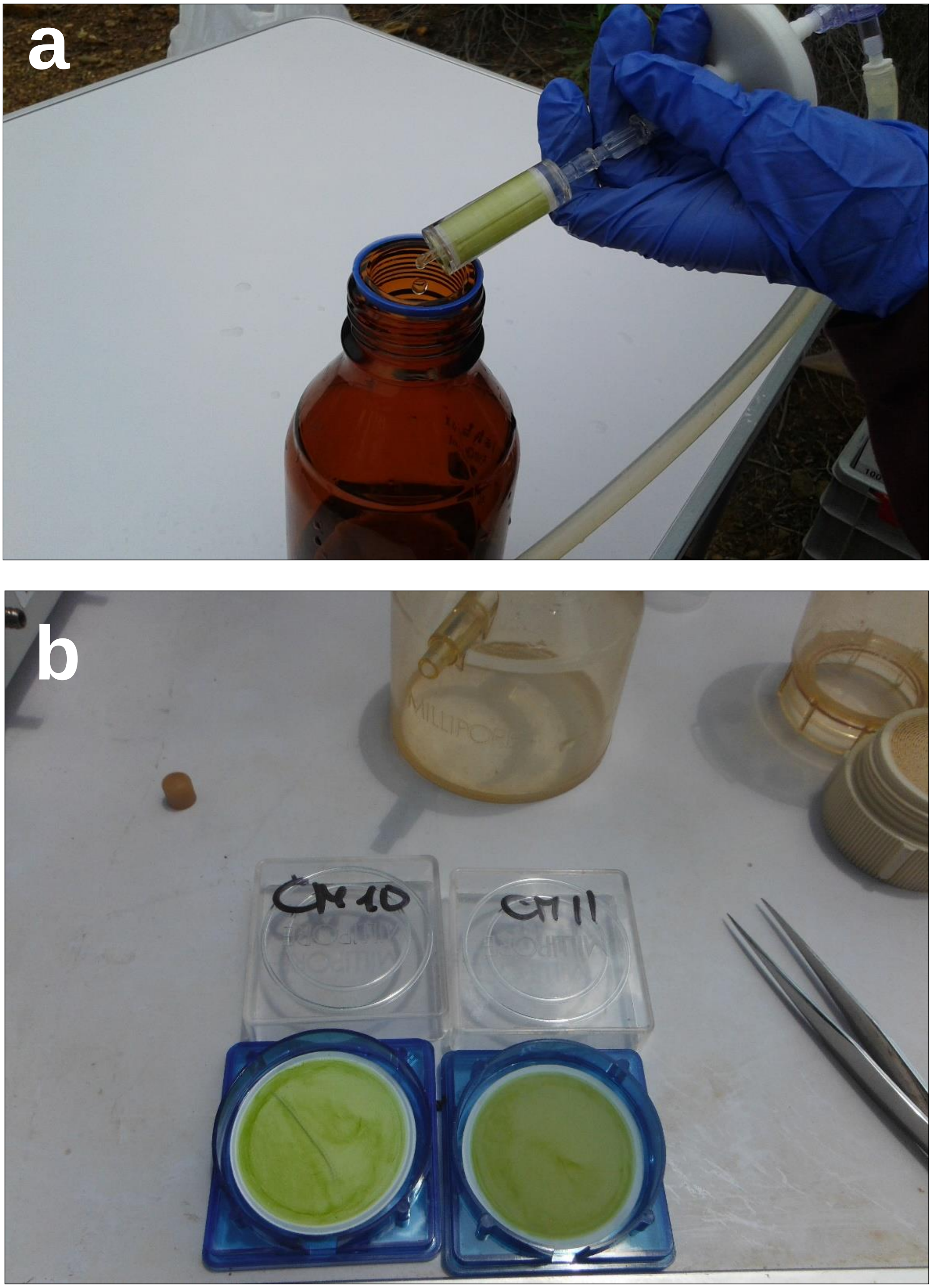

Figure S2. **(a)** Aspect of the Sterivex^{TM} filter (0.22 µm) used to concentrate microbial biomass of phototrophs (for subsequent DNA and RNA extraction for metagenomic and metatranscriptomic analyses) from a sample taken at 11 m depth in Cueva de la Mora acidic pit lake (May 2018). **(b)** Detail of nitrocellulose membrane filters (0.45 µm, Millipore) used to filter water from 10 m and 11 m depths in the same pit lake in July 2020 (photo cortesy of Dr. Iñaki Yusta). The intense green color of the filters denotes abundance of chlorophyll at those depths.

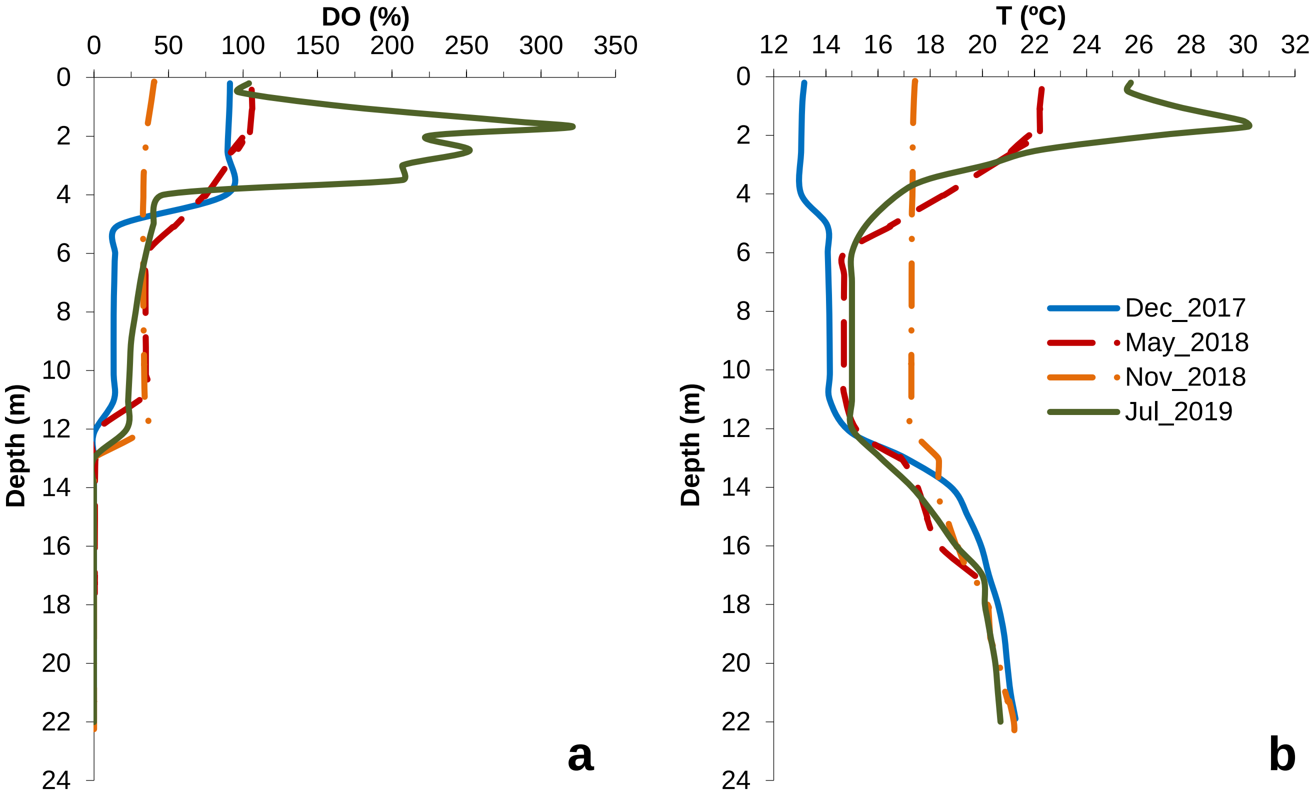

Figure S3. Vertical profiles of dissolved oxygen concentration (DO) **(a)** and temperature (T) **(b)** obtained in different seasons between 2017 and 2019 in the extremely acidic, metal-rich pit lake of Brunita mine (La Unión, SE Spain) (reprinted from [19] with kind permission from Springer Science and Business Media). The outstanding thermal anomaly observed at 2 m below the lake surface in July 2019 (30 ºC *vs*. 26 ºC at the surface) was paralleled by a corresponding DO peak of 325 %sat.

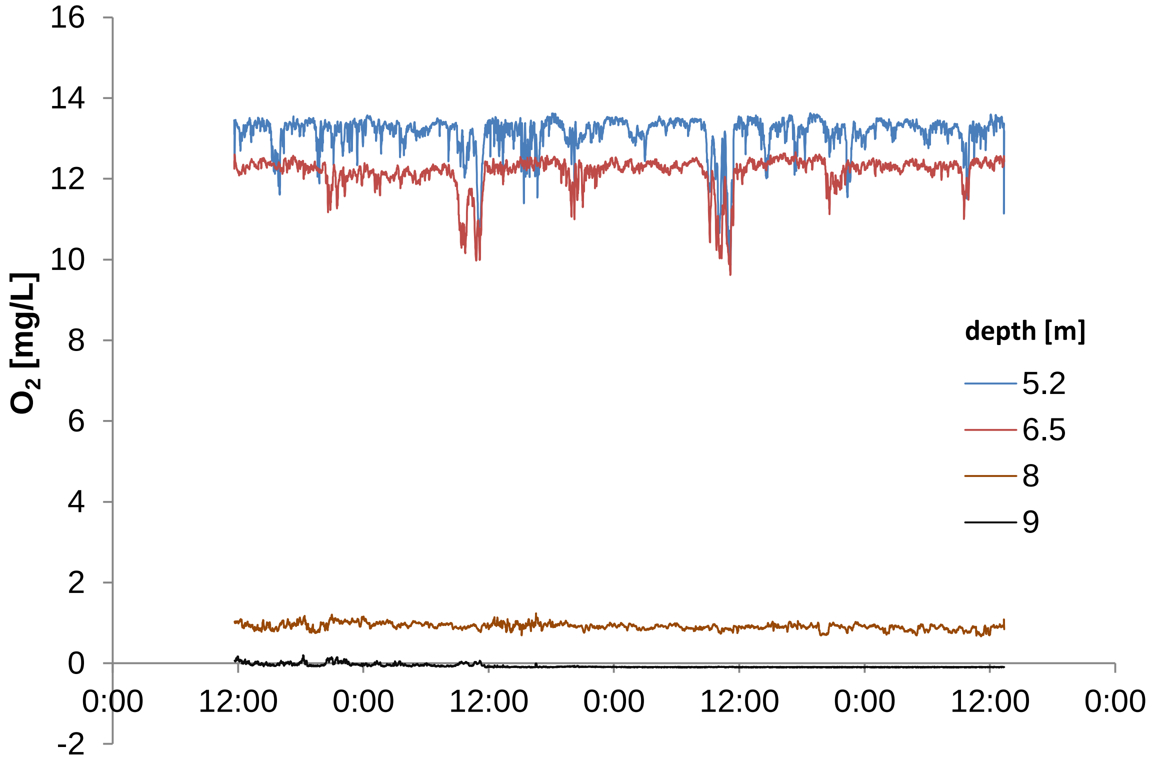

Figure S4. Diel cycles of dissolved oxygen (O_2) concentration measured with data loggers at four different depths (5.2 m, 6.5 m, 8 m and 9 m) in the Cueva de la Mora acidic pit lake during three consecutive days in September 2011.

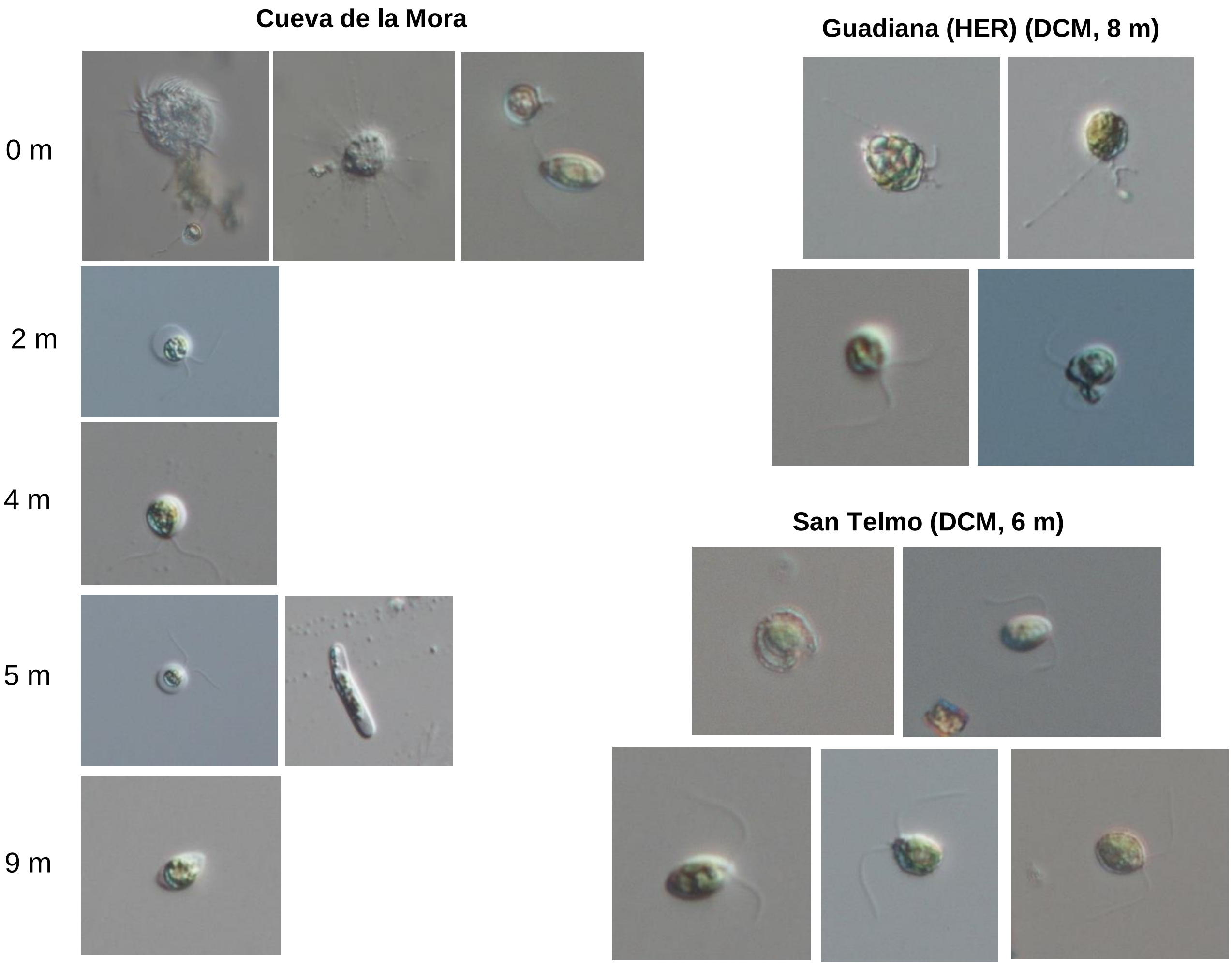

Figure S5. Microscopic images of eukaryotic microorganisms observed at different depths in the APL of Cueva de la Mora (***left***), and in the DCM of Herrerías-Guadiana (***top right***) and San Telmo (***bottom right***) in September 2011. Magnification varies between 100x and 200x. The top left photographs at 0 m corresponded to a ciliate and a heliozoan. The other photographs correspond to unidentified phototrophic microorganisms.

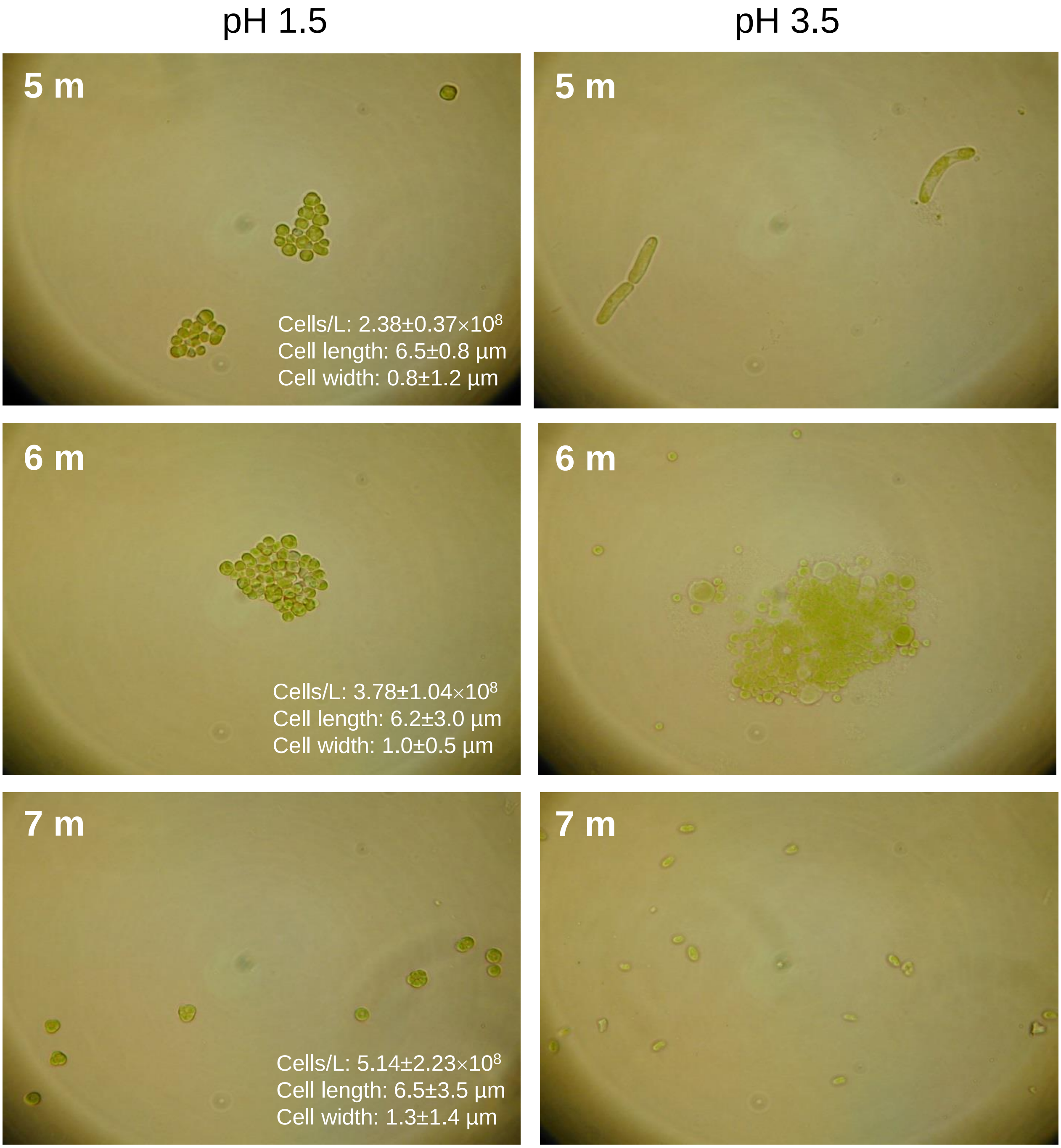

Figure S6. Microscope images of phototrophic microorganisms grown in cultures at room temperature with samples taken from Cueva de la Mora pit lake in September 2017. These samples were taken at depths of 5 m (top), 6 m (center) and 7 m (bottom) from the lake surface, and were grown at pH values of 1.5 (left) and 3.5 (right). The information provided in the pictures of the left column include the number of cells per liter (counted from fixed samples in September 2011), average cell length and average cell width (±standard deviation) measured in the lab in aliquot samples, so that these data correspond to the original conditions found in water samples. The microorganism growing at all depths at pH 1.5 was similar to *Cyanidium caldarium*, whereas those grown at pH 3.5 in the sample from 5 m was an unknown diatom species, and that from 7 m was similar to *Coccomyxa onubensis*. The organism grown at pH 3.5 from 6 m could not be identified. The field of view is 150 µm across in all cases.

HOUSE-KEEPING GENES (replicate 1)

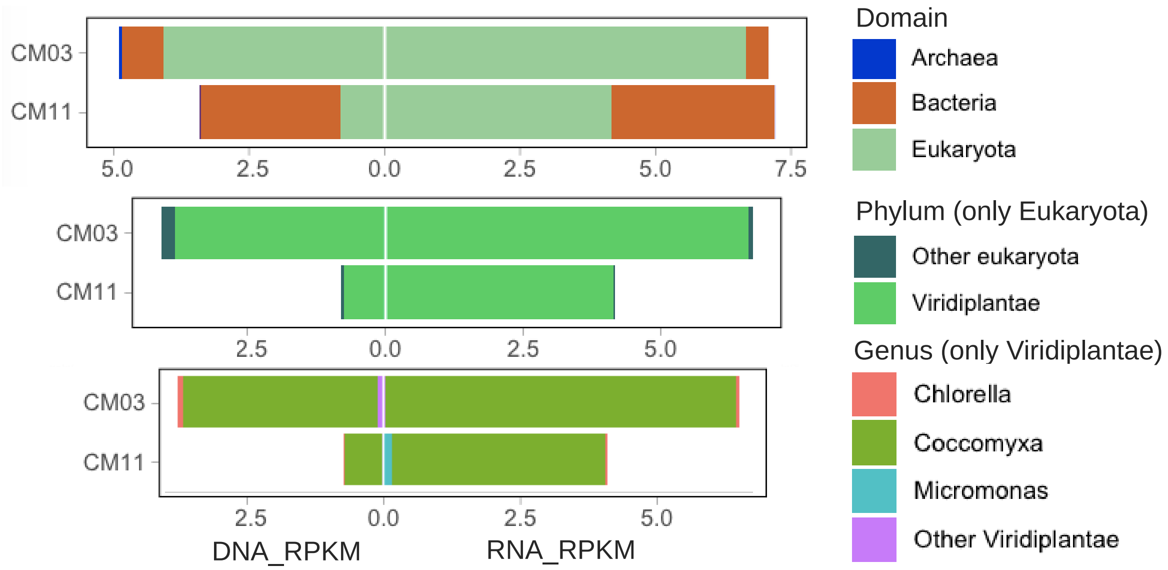

Figure S7. Total frequency (DNA_RPKM) and expression (RNA_RPKM) of predicted genes functionally annotated as house-keeping genes obtained from metagenomic and metatranscriptomic analysis conducted in the APL Cueva de la Mora. RPKM refers to Reads mapped to a predicted gene per Kilobase (length of the predicted gene) per Million Reads (total number of reads mapped to all predicted genes found in the metagenome or metatranscriptome): **(top)** barplot representing the total sum of RPKM values for the predicted genes affiliated to the three different domains; **(center)** same as (top) with the bars broken down by phylum only focused on Eukaryota; **(bottom)** total sum of RPKM values for the predicted genes affiliated to specifically the phylum Viridiplantae (green Algae) at the genus level. ***Note***: replicate 2 is given in Fig. 10 of the manuscript.
